# Paracrine role of endothelial IGF-1 receptor in depot-specific adipose tissue adaptation in male mice

**DOI:** 10.1038/s41467-024-54669-1

**Published:** 2025-01-02

**Authors:** Cheukyau Luk, Katherine I. Bridge, Nele Warmke, Katie J. Simmons, Michael Drozd, Amy Moran, Amanda D. V. MacCannell, Chew W. Cheng, Sam Straw, Jason L. Scragg, Jessica Smith, Claire H. Ozber, Chloe G. Wilkinson, Anna Skromna, Natallia Makava, Hiran A. Prag, T. Simon Futers, Oliver I. Brown, Alexander-Francisco Bruns, Andrew MN Walker, Nicole T. Watt, Romana Mughal, Kathryn J. Griffin, Nadira Y. Yuldasheva, Sunti Limumpornpetch, Hema Viswambharan, Piruthivi Sukumar, David J. Beech, Antonio Vidal-Puig, Klaus K. Witte, Michael P. Murphy, Richard C. Hartley, Stephen B. Wheatcroft, Richard M. Cubbon, Lee D. Roberts, Mark T. Kearney, Natalie J. Haywood

**Affiliations:** 1 Leeds Institute of Cardiovascular and Metabolic Medicine, Faculty of Medicine and Health, University of Leeds, Leeds, UK; 2 MRC Mitochondrial Biology Unit, University of Cambridge, Cambridge Biomedical Campus, Cambridge, UK; 3 University of Cambridge Metabolic Research Laboratories, Cambridge, UK; 4 School of Chemistry, University of Glasgow, Glasgow, UK; 5 Present Address: Integrative Vascular Biology Laboratory, Max Delbrück Center for Molecular Medicine in the Helmholtz Association, Berlin, Germany; 6 Present Address: School of Biomedical Sciences, Faculty of Biological Sciences & Astbury Centre, University of Leeds, Leeds, UK; 7 Present Address: Division of Gastroenterology & Surgery, Leeds Institute of Medical Research, Faculty of Medicine and Health, University of Leeds, Leeds, UK; 8 Present Address: North West Genomic Laboratory Hub, Manchester University NHS Foundation Trust, St Mary’s Hospital, Oxford Road, Manchester, UK; 9 Present Address: Department of Optometry and Vision Sciences, University of Huddersfield, Huddersfield, UK; 10 Present Address: Division of Internal Medicine, Cardiology Unit, Faculty of Medicine Prince of Songkla University, Songkhla, Thailand

**Keywords:** Pre-diabetes, Drug development

## Abstract

During recent decades, changes in lifestyle have led to widespread nutritional obesity and its related complications. Remodelling adipose tissue as a therapeutic goal for obesity and its complications has attracted much attention and continues to be actively explored. The endothelium lines all blood vessels and is close to all cells, including adipocytes. The endothelium has been suggested to act as a paracrine organ. We explore the role of endothelial insulin-like growth factor-1 receptor (IGF-1R), as a paracrine modulator of white adipose phenotype. We show that a reduction in endothelial IGF-1R expression in the presence of high-fat feeding in male mice leads to depot-specific beneficial white adipose tissue remodelling, increases whole-body energy expenditure and enhances insulin sensitivity via a non-cell-autonomous paracrine mechanism. We demonstrate that increased endothelial malonate may be contributory and that malonate prodrugs have potentially therapeutically relevant properties in the treatment of obesity-related metabolic disease.

## Abstract (web summary)

Here the authors show that lowering IGF-1R levels in endothelial cells improves insulin sensitivity, boosts energy use, and supports beneficial changes in adipose tissue, offering insights into potential treatments for obesity-related metabolic disorders.

## Introduction

Over the past four decades, changes in human lifestyle have contributed to a pandemic of nutritional obesity^1^. Obesity occurs due to sustained elevation of calorie intake, most often in the form of lipids and carbohydrates, and/or a decline in energy expenditure^2^. Disruption of this ‘energy balance equation'^3^ can occur at any point during human life. In 2015, over 100 million children and 600 million adults were living with obesity worldwide^4^. An unfavourable deviation in the energy balance equation in favour of calorie excess results in increased adiposity and eventually ectopic deposition of lipids in tissues such as the liver and skeletal muscle, which are ill-equipped to deal with this challenge. As a result, deleterious perturbations in cellular function increase the risk of type 2 diabetes mellitus, accelerated cardiovascular disease, fatty liver and some cancers^5^ (for review).

Dietary lipids are stored in adipose tissue (AT). There are two types of AT; White AT (WAT), which is specialised in storing energy in the form of triglyceride and undergoes expansive remodelling during nutrient excess with adipocytes adopting a hypertrophic/hyperplastic phenotype^6^. The second form of AT is brown AT (BAT)^7^. BAT, unlike WAT, expresses the mitochondrial uncoupling protein-1 (UCP-1). UCP-1 uncouples cellular respiration from mitochondrial ATP synthesis, affording BAT the capacity to oxidise lipids and glucose to generate heat^7^.

Historically, WAT was thought to be a simple storage depot for lipids. However, over the past two decades, research has revealed WAT to be a complex and plastic organ (Reviewed^8^). In obesity, the relationship between WAT expansion and neovascularisation becomes uncoupled, leading to inadequate perfusion of adipocytes^9^. Under these circumstances, WAT becomes dysfunctional, and the secretory profile of adipocytes becomes unfavourable, metabolically detrimental, and contributes to increased cardiovascular risk^10,11^. Therefore, targeting WAT to mitigate against the adverse sequelae of obesity has received significant attention^12–17^. Mechanisms of changing WAT from a storage to oxidative/thermogenic phenotype have been of particular interest^18–21^.

The insulin/insulin-like growth factor-1 (IGF-1) signalling system evolved millions of years ago to co-ordinate organismal growth and metabolism. During evolution, the insulin receptor (IR) and IGF-1 receptor (IGF-1R) diverged from a single receptor in invertebrates into a more complex system in mammals consisting of the IR, IGF-1R and their respective ligands; insulin, IGF-1 and IGF-II (^22^ for our review). We have previously shown that circulating IGF-1 increases and IGF-1R levels decline during calorie excess in various tissues, including the vasculature^23,24^.

The endothelium lines all blood vessels and may act as a paracrine organ^25–31^, including endothelial to AT signalling^32–34^. Here, we explored the role of endothelial IGF-1R as a paracrine modulator in the pathophysiology of obesity. We have discovered that endothelial cell-derived malonate, prevents deleterious remodelling of WAT, presenting therapeutic opportunities.

## Results

### Murine endothelial IGF-1R knockdown enhances insulin sensitivity

**Fig. 1:**
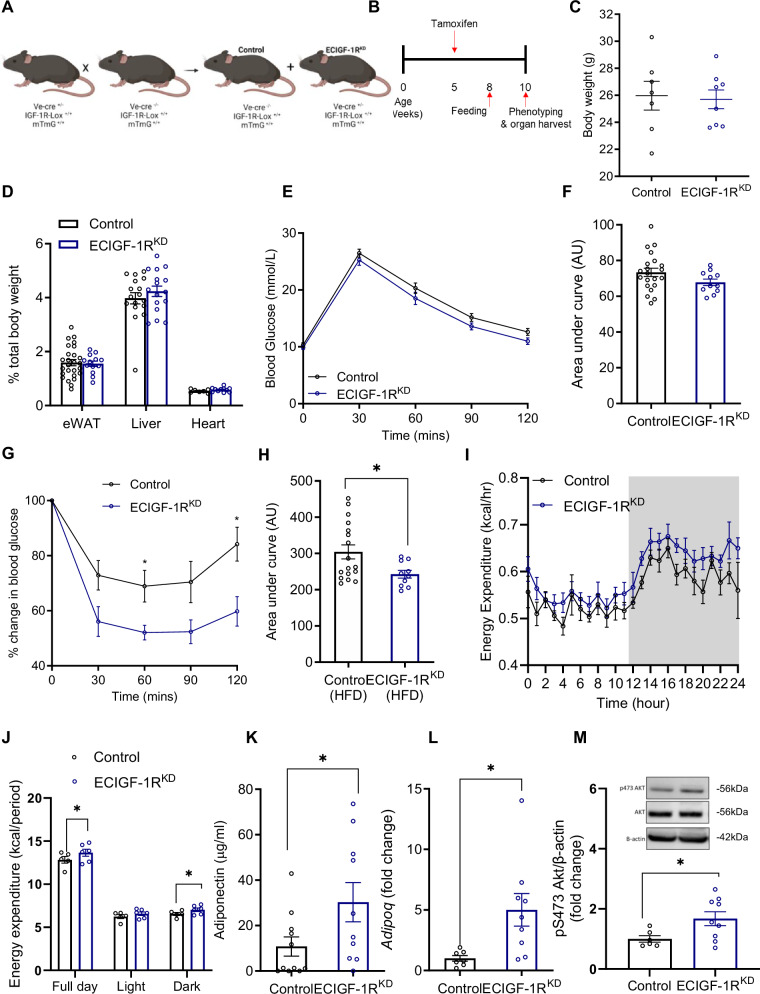
Reduction in murine endothelial IGF-1R expression improves whole body insulin sensitivity and energy expenditure in the setting of high fat feeding. **A** Schematic representation of the generation of tamoxifen-inducible endothelial cell specific IGF-1R knockdown mice (ECIGF-1R^KD^). Created in BioRender. Luk, C. (2023) https://BioRender.com/x35a412. **B** Schematic representation of experimental protocol. **C** Quantification of body weight from 2-week high fat fed (HFD) control and ECIGF-1R^KD^ mice. (*n* = 7 and 8). **D** Quantification of wet organ weight from 2-week HFD control and ECIGF-1R^KD^ mice (*n* = eWAT 26 and 13, Liver 17 and 16 and Heart 7 and 11). **E** Glucose tolerance over time for 2-week HFD control and ECIGF-1R^KD^ mice (*n* = 21 and 11). **F** Area under the curve (AUC) analysis for glucose tolerance for 2-week HFD control and ECIGF-1R^KD^ mice (*n* = 21 and 11). **G** Insulin tolerance test for 2-week HFD control and ECIGF-1R^KD^ mice (*n* = 17 and 10). *p* = 0.04 and 0.01 for 60 min and 120 min respectively. **H** The area under the curve analysis for insulin tolerance tests for 2-week HFD control and ECIGF-1R^KD^ mice (*n* = 17 and 10). *p* = 0.03. **I** Energy expenditure in 2-week HFD fed control and ECIGF-1R^KD^ mice over 24 h period (*n* = 4 and 6). The dark cycle is shown in grey. **J** Average energy expenditure in 2-week HFD fed control and ECIGF-1R^KD^ mice during specific time periods (*n* = 4 and 6). *p* = 0.04 and 0.03 for full day and dark cycle respectively. **K** Quantification of plasma adiponectin levels from 2-week HFD control and ECIGF-1R^KD^ mice (*n* = 10 and 11). *p* = 0.05. **L** Quantification of Brown adipose tissue adiponectin gene expression from 2-week HFD control and ECIGF-1R^KD^ mice (*n* = 7 and 8). *p* = 0.02. **M** Quantification and representative images of western blots for p473 AKT, AKT and B-actin from muscle from 2-week HFD fed control and ECIGF-1R^KD^ mice (*n* = 6 and 9). *p* = 0.04. Data shown as mean ± SEM, data points are individual mice. Metabolic parameters were measured by indirect calorimetry, ANOVA testing was performed using calrapp.org. *p* < 0.05 taken as statistically significant using student unpaired two-tailed *t*-test and denoted as *.

To investigate the role of endothelial IGF-1R in the setting of increased energy balance, we generated a tamoxifen-inducible, endothelial cell-specific IGF-1R knockdown mouse (ECIGF-1R^KD^) (Fig. 1A, B) with an mTmG reporter to confirm spatially appropriate Cre-recombinase activity (Supplementary Fig. 1A–C). When unchallenged on a standard laboratory chow diet, ECIGF-1R^KD^ mice exhibited no difference in body weight (Supplementary Fig. 1D). Glucose and insulin tolerance were also unchanged in chow-fed mice (Supplementary Fig. 1E–H).

When challenged with a high-fat diet (HFD) for two weeks, ECIGF-1R^KD^ mice had comparable body and organ weight (Fig. 1C, D) and similar glucose tolerance to their control littermates (Fig. 1E, F). However, insulin sensitivity (Fig. 1G, H) and energy expenditure (Fig. 1I, J) were greater in ECIGF-1R^KD^ after HFD feeding for two weeks, compared to control littermates on the same diet (Fig. 1G, H). Higher energy expenditure was associated with increased carbon dioxide production (Supplementary Fig. 2A, B), increased oxygen consumption (Supplementary Fig. 2C, D) and increased food consumption during the dark phase of their light-dark cycle (Supplementary Fig. 2E, F), but without change in activity levels (Supplementary Fig. 2G, J).

After two weeks of HFD, ECIGF-1R^KD^ mice had similar core body temperature (Supplementary fig. 3A) and fasting plasma concentrations of glucose, insulin, IGF-I, free fatty acids, triglycerides, and leptin as control littermates on the same diet (Supplementary fig. 3B–G). ECIGF-1R^KD^ mice had increased circulating levels of the beneficial adipokine adiponectin and increased adiponectin gene expression in BAT (Fig. 1K, L) along with increased levels of phosphorylation of AKT at its activation site serine 473 in muscle (Fig. 1M).

### Endothelial IGF-1R knockdown halts deleterious adipose remodelling

**Fig. 2:**
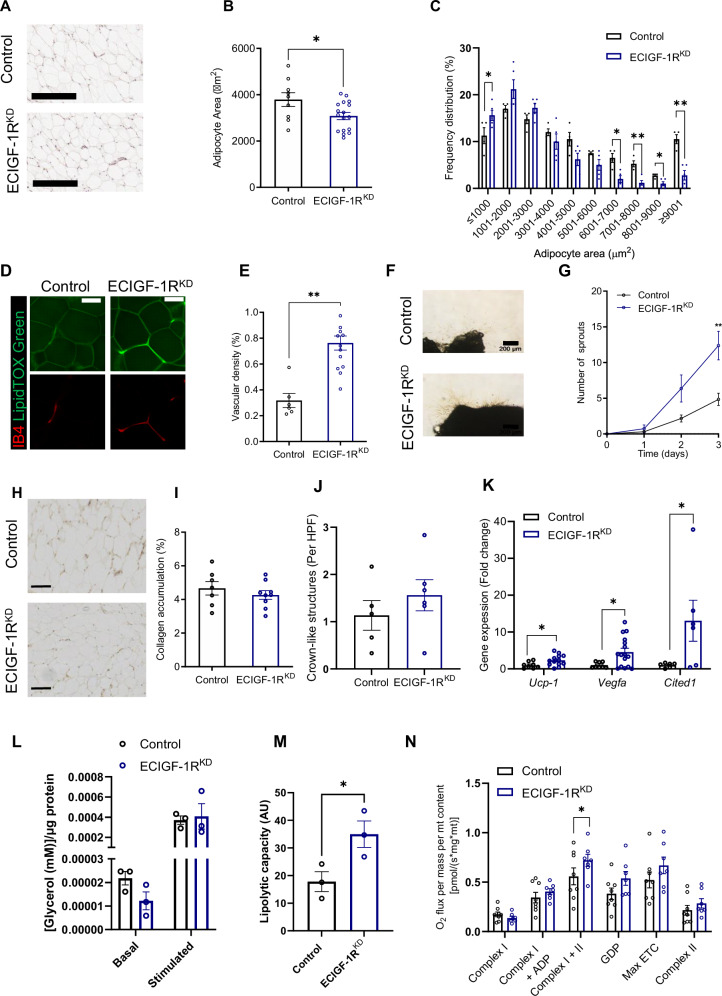
Reduction in murine endothelial IGF-1R expression prevents deleterious remodelling of epididymal white adipose tissue in the setting of high fat feeding. **A** Representative images of hematoxylin and eosin (H and E) stained epididymal white adipose tissue (eWAT) from 2-week high fat feeding (HFD) control and tamoxifen-inducible endothelial cell specific IGF-1R knockdown mice (ECIGF-1R^KD^) mice (Scale bar = 200 µm). **B** Quantification of adipocyte size in eWAT from 2-week HFD control and ECIGF-1R^KD^ mice (*n* = 9 and 19). *p* = 0.02. **C** Quantification of eWAT adipocyte size distribution from 2-week HFD control and ECIGF-1R^KD^ mice (*n* = 9 and 19). *p* = 0.05, 0.006, 0.001, 0.01, and 0.001 for ranges ≤1000, 6001–7000,7001–8000, 8001–9000 and >9000 respectively. **D** Representative images of isolectin B4 (IB4, Red) and LipidTox (Green) stained eWAT from 2-week HFD control and ECIGF-1R^KD^ mice (Scale bar = 100 µm). **E** Quantification of eWAT vascularisation from 2-week HFD control and ECIGF-1R^KD^ mice (*n* = 6 and 14). *p* = 0.001. **F** Representative images of 2-week HFD control and ECIGF-1R^KD^ eWAT tissue explants (Scale bar = 200 µm). **G** Quantification of eWAT neovascularisation from 2-week HFD control and ECIGF-1R^KD^ mice (*n* = 5 and 5). *p* = 0.008. **H** Representative images of picro sirius red stained eWAT from 2-week HFD control and ECIGF-1R^KD^ mice (Scale bar = 200 µm). **I** Quantification of eWAT collagen deposition from 2-week HFD control and ECIGF-1RKD mice (*n* = 7 and 9). **J** Quantification of eWAT crown like structures from 2-week HFD control and ECIGF-1RKD mice per high powered field (HPF) (*n* = 5 and 6). **K** Quantification of eWAT upregulated gene expression from 2-week HFD control and ECIGF-1R^KD^ mice (Ucp1 *n* = 8 and 13, Vegfa 8 and 16, Cited1 6 and 6). *p* = 0.03, 0.02 and 0.05 for Ucp1, Vegfa and cited 1 respectively. **L** Quantification of basal and forskolin stimulated extracellular glycerol concentration from eWAT from 2-week HFD control and ECIGF-1R^KD^ mice (*n* = 3 and 3 mice and 5 explants per mouse). **M** Quantification of lipolytic capacity from eWAT from 2-week HFD control and ECIGF-1R^KD^ mice (*n* = 3 and 3 mice and 5 explants per mouse). *P* = 0.05. **N** Quantification of respiration from eWAT normalised to mitochondrial content from 2-week HFD control and ECIGF-1R^KD^ mice (*n* = 7 and 8). *p* = 0.05. Data shown as mean ± SEM, data points are individual mice. *p* < 0.05 taken as statistically significant using student unpaired two tailed *t*-test and denoted as * (**p ≤ 0.01). For respirometry data two-way ANOVA corrected for multiple comparisons by controlling the False Discovery Rate using two-stage step-up method of Benjamani, Krieger and Yekutieli was used.

After two weeks of HFD, epididymal WAT (eWAT) from ECIGF-1R^KD,^ mice had smaller adipocytes (Fig. 2A–C), increased vascularity (Fig. 2D, E) and enhanced ex vivo sprouting angiogenesis (Fig. 2F, G, Supplementary Fig. 4) compared to their control littermates. There was no difference in eWAT vascularity between chow-fed ECIGF-1R^KD^ mice and their littermate controls on the same diet (Supplementary Fig. 1I, J), indicating a specific response to high-fat feeding. There was no change in eWAT fibrosis (Fig. 2H, I) and no change in macrophage infiltration, shown by crown‐like structure (CLS) analysis (Fig. 2J), after two weeks of HFD between the genotypes.

ECIGF-1R^KD^ mice fed a HFD for 2 weeks had increased expression of *Ucp1, Vegfa* and *Cited* (Fig. 2K) and downregulation of *Tmem28* and *Fgf21* (Supplementary Fig. 5A) in eWAT, whilst various other genes involved in adipocyte function and were unchanged (Supplementary Fig. 5B), when compared to their control littermates on the same diet. On chow diet, *Ucp1* and *Vegfa* eWAT gene expression levels in chow-fed ECIGF-1R^KD^ mice were unchanged compared to control mice (Supplementary Fig. 1K).

eWAT explants from ECIGF-1R^KD^ mice fed HFD for 2 weeks, had a trend towards reduced basal lipolysis and no change in forskolin-stimulated lipolysis (Fig. 2L), resulting in greater lipolytic capacity (Fig. 2M), when compared to eWAT explants from control mice. High-resolution respirometry revealed eWAT from ECIGF-1R^KD^ mice fed a HFD for 2 weeks had increased complex I and II mediated respiration (Fig. 2N). Taken together, we show that reduced endothelial IGF-1R expression in the presence of high-fat feeding prevents adverse eWAT remodelling, increases whole-body energy expenditure, and enhances insulin sensitivity.

### Endothelial IGF-1R knockdown effects are adipose depot-specific

Next, we characterised another WAT depot from ECIGF-1R^KD^ mice fed a HFD for two weeks by exploring inguinal adipose tissue (iWAT), which is a depot traditionally responsive to beiging. iWAT mass (Supplementary Fig. 6A), adipocyte size (Supplementary Fig. 6B–D), crown-like structures (Supplementary Fig. 6E) and vascularity (Supplementary Fig. 6F, G) were not different in ECIGF-1R^KD^ mice when compared to control littermates on HFD. There was also no change in mitochondrial respiration of this depot (Supplementary Fig. 6H), and expression of various genes involved in adipose function in iWAT was also unchanged (Supplementary Fig. 6I).

ECIGF-1R^KD^ mice had similar lipid accumulation in the liver and interscapular BAT (Supplementary Fig. 7A–D). Vascularity in other AT depots (BAT and perinephric WAT) was unchanged (Supplementary Fig. 7E–H), as well as vascularity of other organs, including the liver and muscle (Supplementary Fig. 7I–L), suggesting that the effect of reduced endothelial IGF-1R is specific to eWAT.

### Endothelial IGF-1R knockdown persist after long-term feeding

To explore the chronicity of these findings, a separate cohort of ECIGF-1R^KD^ mice and littermate controls received HFD for eight weeks. ECIGF-1R^KD^ mice maintained comparable body weight, organ weight, and glucose tolerance compared to control littermates (Supplementary Fig. 8A–D). The enhanced insulin sensitivity of ECIGF-1R^KD^ seen at 2 weeks HFD was present, although not as sustained during tolerance testing, after 8 weeks of HFD (Supplementary Fig. 8E, F). Circulating insulin, IGF-I and adiponectin concentrations were unchanged (Supplementary Fig. 8G–I). eWAT from ECIGF-1R^KD^ mice after eight weeks HFD had smaller adipocytes (Supplementary Fig. 9A, B). At this time point, ECIGF-1R^KD^ mice also had reduced lipid accumulation in BAT (Supplementary Fig. 9C, D). There was no longer a difference in eWAT vascularity (Supplementary Fig. 9E, F); but increased *Ucp1* and *Vegfa* gene expression was retained (Supplementary Fig. 9G). In BAT increased gene expression on adiponectin was lost (Supplementary Fig. 9H). There was no difference in eWAT collagen deposition (Supplementary Fig. 9I, J), crown-like structures (Supplementary Fig. 9K) or fatty liver in ECIGF-1R^KD^ mice after 8 weeks HFD compared to control littermates (Supplementary Fig. 9L, M). These findings suggest that the advantageous effects of endothelial cell IGF-1R knockdown on whole-body insulin sensitivity and eWAT phenotype are retained after prolonged HFD exposure.

### Endothelial IGF-1R knockdown alters adipocyte paracrine modulation

To probe mechanisms underpinning the favourable changes to eWAT in ECIGF-1R^KD^ mice receiving HFD, we investigated the possibility that adipocytes were directly derived from ECIGF-1R^KD^ endothelial cells, as it has been demonstrated that adipocytes of endothelial origin exist in BAT and WAT^35^. However, in our model, Cre activity in ECIGF-1R^KD^ resulted in vascular GFP expression, as intended, but no GFP expressing adipocyte-like structures were observed after 2 weeks of HFD, showing endothelial cell to adipocyte transformation was not occurring (Supplementary Fig. 10).

**Fig. 3:**
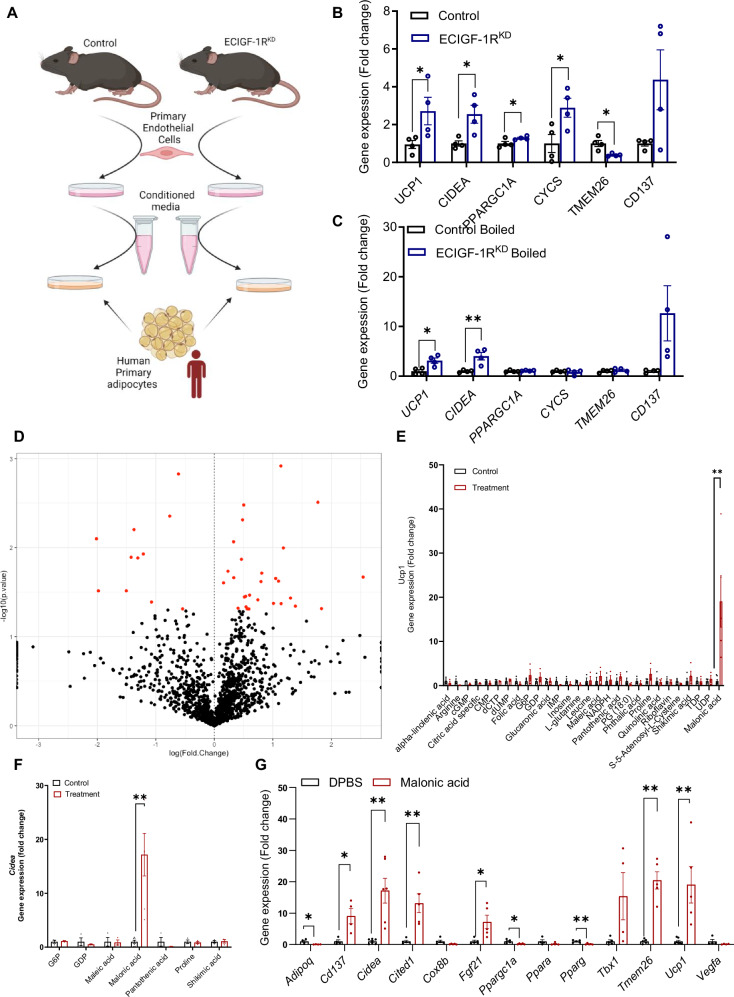
Reduction in murine endothelial IGF-1R expression alters the endothelial secretome and reveals a role for malonate in modulating white adipose function. **A** Schematic representation of conditioned media experimental protocol. Created in BioRender. Luk, C. (2023) https://BioRender.com/x03i976. **B** Quantification of human primary adipocyte gene expression after 24 h treatment with conditioned media from primary murine endothelial cells isolated from 2-week high fat feeding (HFD) control and tamoxifen-inducible endothelial cell specific IGF-1R knockdown mice (ECIGF-1R^KD^) mice (*n* = 4 and 4). *p* = 0.05, 0.01, 0.05, 0.03 and 0.01 for Ucp1, CIDEA, PPARGC1A, CYCS, TMEM26. **C** Quantification of human primary adipocyte gene expression after treatment with boiled conditioned media from primary murine endothelial cells isolated from 2-week HFD fed control and ECIGF-1R^KD^ mice (*n* = 4 and 4). *p* = 0.01 and 0.007 for Ucp-1 and Cidea respectively. **D** Volcano plot of small molecule analysis of the aqueous and lipid fractions of conditioned media from primary murine endothelial cell from 2-week HFD fed control and ECIGF-1R^KD^ mice (*n* = 4 and 4 per genotype). Red dots are significantly different between the genotypes. **E** Quantification of 3T3-L1 adipocyte gene expression of Ucp1 after upregulated metabolite stimulation (*n* = 3 and 3 for all treatment groups apart from PG, Quinolinic acid and shikimic acid *n* = 4 and 4 and malonic acid *n* = 7 and 5). *p* = 0.004. **F** Quantification of 3T3-L1 adipocyte gene expression of Cidea after upregulated metabolite stimulation (*n* = 3 and 3 per treatment group expect malonic acid *n* = 7 and 6). *p* = 0.001. **G** Quantification of gene expression in 3T3-L1 adipocytes after 24 h 10 mM malonic acid stimulation (*n* = 5 and 5 per gene except, Adipoq, Cd137, Ppara, Vegfa *n* = 4 and 4, Tbx1 *N* = 5 and 4, Cidea *N* = 7 and 6 and Ucp-1 *n* = 7 and 5). *P* = 0.03, 0.01, 0.00.1,0.004, 0.02, 0.01, 0.001, 0.0001, and 0.003 for Adipoq, Cd137, Cidea, Cited1, Fgf21, Ppargc1a, Pparg, Tmem26, Ucp1 and Vegfa respectively. Data shown as mean ± SEM, *n* is an individual experiment. *p* < 0.05 taken statistically significant using student unpaired two tailed *t*-test and denoted as * (*p* ≤ 0.01 and is denoted as **). For 3D an independent parametric Welch *t*-test, assuming two-group variances may differ was used. The statistical significance was determined by *P*-value with no multiple corrections.

Since the endothelium may function as a paracrine organ^25–28^, we investigated a potential paracrine mechanism facilitating cross-talk between endothelium and eWAT (Fig. 3A). Treatment of primary human adipocytes with conditioned media from primary endothelial cells of ECIGF-1R^KD^ fed HFD for two weeks led to increased *UCP1*, *CIDEA*, *PPARGC1A*, *CYCS* and *CD137* gene expression compared to human adipocytes cultured in conditioned media from endothelial cells of control littermates fed HFD for two weeks (Fig. 3B). *UCP1* and *CIDEA* expression induced by ECIGF-1R^KD^ EC conditioned media was preserved following protein denaturation by boiling, suggesting a non-protein paracrine signal (Fig. 3C). We used an unbiased metabolomic approach to compare the small molecule secretome of primary endothelial cells from ECIGF-1R^KD^ mice and their littermate controls after two weeks of HFD and found distinct differences in the small molecule endothelial secretome, including a 60% increase in malonate (Fig. 3D).

### Malonate is a adipose tissue modulator

A screen of the upregulated metabolites released from ECIGF-1R^KD^ endothelial cells (Fig. 3E, F) revealed that malonic acid was sufficient to upregulate *Ucp1, Cidea, Cd137, Cited1, Fgf21* and *Tmem26* gene expression in 3T3-L1 adipocytes (Fig. 3G). 10 mM malonic acid also increased adiponectin secretion from 3T3-L1 adipocytes (Supplementary Fig. 11A) and glucose uptake in 3T3-L1 adipocytes (Supplementary Fig. 11B). Time-course experiments demonstrated malonic acid upregulated *Fgf21* gene expression, followed by *Ucp1* and *Cidea* gene expression (Supplementary Fig. 11C).

Inhibition of succinate dehydrogenase (SDH) by malonate can alter mitochondrial redox status, potentially altering mitochondrial signallling pathways^36^. We, therefore, treated adipocytes with MitoQ, a mitochondria-targeted antioxidant^37^, prior to malonic acid treatment. MitoQ attenuated malonic acid-induced *Fgf21, UCP1 and Cidea* gene upregulation (Supplementary Fig. 11D), suggesting that malonic acid-induced upregulation of FGF21 signalling is partly dependent on malonate altering mitochondrial redox signalling.

FGF21 was previously reported as a paracrine/autocrine beiging mediator in WAT, enriched in murine rosiglitazone-stimulated beige adipocytes and norepinephrine-stimulated brown adipocytes^38–41^. An FGF21 receptor blocker (Supplementary Fig. 11E) diminished malonic acid-induced upregulation of *Ucp1* and *Cidea* in 3T3-L1 adipocytes, consistent with previous studies showing FGF21 regulates *Ucp1*^42,43^.

Although the action of malonate to inhibit SDH was established over 80 years ago^44^, our data demonstrate that, using an alternative concentration and exposure time, malonate leads to remodelling of WAT. In a shivering thermogenesis model, Mills et al.^45^, suggested that elevated succinate led to browning of WAT in a SDH and ROS-dependent fashion with malonate inhibiting SDH to block the effect of succinate. However, our data raise the possibility that malonate may act to modulate adipocytes in eWAT in an FGF21-dependent fashion possibly through elevated redox signalling (Supplementary Fig. 11F).

### A malonate prodrug is an adipose tissue modulator in obesity

**Fig. 4:**
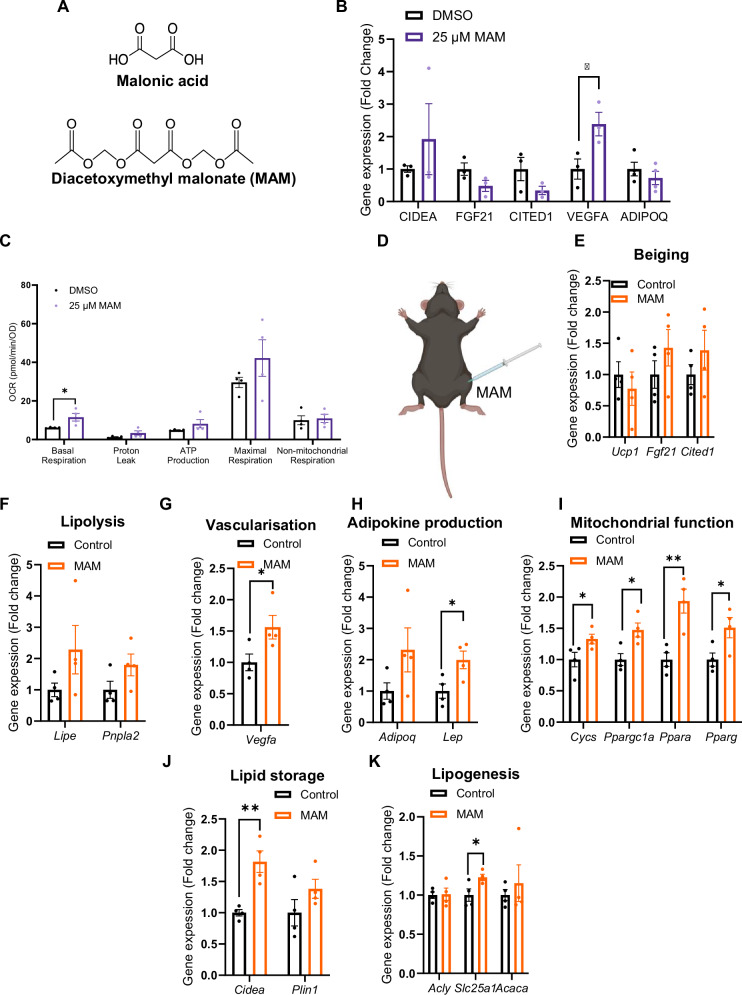
Therapeutic potential of malonate on adipose tissue function. **A** Structural representation of malonic acid and Diacetoxymethyl malonate (MAM) used in vitro and in vivo. **B** Quantification of gene expression in human primary adipocytes after 24 h 25 µM MAM stimulation (*n* = 3 per treatment group). *p* = 0.04. **C** Quantification of respirometry in human primary adipocytes after 24 h 25 µM MAM stimulation (*n* = 4 per treatment group). *p* = 0.03. **D** Schematic representation of location of intra-epididymal adipose delivery of MAM (16 mg/kg). Created in BioRender. Luk, C. (2023) https://BioRender.com/b50h998. **E** Quantification of beiging gene expression from epididymal white adipose tissue (eWAT) from obese mice treated with MAM 16 mg/kg (*n* = 4 per treatment group). **F** Quantification of lipolysis gene expression from eWAT from obese mice treated with MAM 16 mg/kg (*n* = 4 per treatment group). **G** Quantification of vascularisation gene expression from eWAT from obese mice treated with MAM 16 mg/kg (*n* = 4 per treatment group). *p* = 0.03. **H** Quantification of adipokine production gene expression from eWAT from obese mice treated with MAM 16 mg/kg (*n* = 4 per treatment group). *p* = 0.01 for Lep. **I** Quantification of mitochondrial function gene expression from eWAT from obese mice treated with MAM 16 mg/kg (*n* = 4 per treatment group). *p* = 0.05, 0.01, 0.005 and 0.03 for Cycs, Ppargc1a, ppara, ppary respectively. **J** Quantification of lipid storage gene expression from eWAT from obese mice treated with MAM 16 mg/kg (*n* = 4 per treatment group). *p* = 0.04. **K** Quantification of lipogenesis gene expression from eWAT from obese mice treated with MAM 16 mg/kg (*n* = 4 per treatment group). *p* = 0.04. Data shown as mean ± SEM, n is an individual experiment. *p* < 0.05 taken statistically significant using student unpaired two tailed t-test and denoted as * (*p* ≤ 0.01 and is denoted as **). For respirometry data a two-way ANOVA corrected for multiple comparisons by controlling the False Discovery Rate using two-stage step-up method of Benjamani, Krieger and Yekutieli, *p < 0.05.

To explore the therapeutic potential of malonate as an adipose modulator, we utilised diacetoxymethyl malonate (MAM)^46^ (Fig. 4A), which accelerates the malonate delivery, compared to malonic acid due to enhanced membrane permeation under physiological conditions^46^ in a human adipocyte model. MAM induced an upregulation of VEGFA gene expression in primary human adipocytes (Fig. 4B) and basal respiration was also increased in primary human adipocytes with MAM stimulation (Fig. 4C).

We next examined the potential therapeutic capacity of MAM in vivo in the obesity setting. For this purpose, we administered MAM, directly into eWAT of obese wildtype mice (Fig. 4D). An epididymal MAM injection of 16 mg/kg did not affect beiging genes (Fig. 4E) and lipolysis genes (Fig. 4F). However, MAM treatment upregulated various genes involved in adipose function, including those involved in vascularity (Fig. 4G), adipokine production (Fig. 4H), mitochondrial function (Fig. 4I), lipid storage (Fig. 4J) and lipogenesis (Fig. 4K).

## Discussion

Over the past decades, changes in human behaviour, such as increased calorie consumption and sedentary lifestyles, have contributed to increased obesity^1^. As a result, there has been a global increase in the prevalence of type 2 diabetes mellitus, accelerated cardiovascular disease, fatty liver and some cancers^5^, and there is now an urgent need for novel therapeutic strategies to treat obesity and its associated complications.

Initially, WAT was considered a simple storage unit for fat. However, research spanning the last few decades has unveiled WAT’s intricate and adaptable nature^8^. In cases of obesity, the association between the expansion of WAT and neovascularisation becomes disrupted, resulting in insufficient perfusion of adipoctyes^9^. In such scenarios, WAT becomes dysfunctional, and the secretory profile of adipocytes turns metabolically unfavourable^10,11^. Consequently, significant attention has been directed towards targeting the phenotype of WAT to counteract the adverse consequences of obesity^12–21^.

Historically, the endothelium was viewed only as cells forming the blood vessels’ inner lining. However, the endothelium is now emerging as a potentially critical paracrine organ^25–34^. We used a murine model of high-fat feeding with endothelial-specific IGF-1R knockdown to examine the role of this evolutionarily preserved transmembrane receptor in endothelial cells in response to nutritional obesity. When compared to littermate controls on HFD, we found that these mice had enhanced whole-body insulin sensitivity and energy expenditure and increased circulating levels of the beneficial adipokine, adiponectin.

We now know that WAT undergoes a continuous process of remodelling that is pathologically accelerated and dysfunctional in obesity^47^. Here, we report that endothelial IGF-1R knockdown in the setting of obesity blunts the deleterious remodelling of eWAT (the equivalent of the visceral depot in humans) in response to high fat feeding with smaller adipocytes, generally considered a healthier phenotype than larger adipocytes, contributing to insulin resistance^14,48–52^.

We also found that endothelial IGF-1R knockdown in the setting of obesity increased eWAT vascularity, which was associated with increased ex vivo sprouting and *Vegfa* gene expression. This shows an increase in eWAT neovascularisation, preventing AT hypoperfusion, a hallmark of WAT dysfunction^53–57^.

Alterations in lipolysis are associated with obesity, including an increase in basal rate of lipolysis that contributes to the development of insulin resistance, as well as impaired responsiveness to stimulated lipolysis^58^. We report that eWAT from mice with endothelial IGF-1R knockdown had a strong trend for reduced basal levels of lipolysis compared to control mice and had a significantly higher lipolytic capacity. Further research is warranted to investigate the mechanisms underlying the enhanced lipolysis seen in eWAT.

Although the primary focus of this manuscript is on eWAT, it is worth mentioning that we also observed an increase in circulating plasma levels of the beneficial cytokine adiponectin. This finding was accompanied by elevated adiponectin gene expression in BAT. Therefore, future studies are warranted to investigate the role of the IGF-1R in endothelial-AT crosstalk, particularly in BAT.

Recent studies indicate that at least two types of thermogenic adipocyte exist in mammals: a pre-existing form established during development, termed ‘classical brown’ and an inducible form described as ‘beige’^59,60^. BAT depots previously thought to be limited to neonates have been identified in human adults^61–63^. Beige adipocyte biogenesis can be stimulated by various environmental cues such as chronic cold exposure^59,60^, leading to a process frequently referred to as ‘beiging’ of WAT. The potentially favourable metabolic effects of increasing thermogenic AT during increased energy balance have led investigators to explore different approaches to stimulate WAT beiging. In addition to the beneficial WAT remodelling discussed above, mice with endothelial IGF-1R knockdown also had significant changes in thermogenic/beiging gene expression, including an upregulation of uncoupling protein-1 (*Ucp-1)*. Thermogenic AT, unlike pure WAT, expresses *UCP-1*, which uncouples cellular respiration from mitochondrial ATP synthesis, affording thermogenic AT the ability to burn fat^7^ and reduce weight gain in the presence of increased energy balance.

Therefore, we examined the cellular respiration of eWAT. To first determine if UCP-1-mediated respiration was altered in mice with endothelial IGF-1R knockdown, we used the UCP-1 inhibitor GDP^64^. Although mice with endothelial IGF-1R knockdown had higher *Ucp1* gene expression, there was no difference in UCP-1-mediated oxygen consumption in eWAT. Substances and inhibitors of each complex were used to determine if respiration mediated by other mitochondrial respiration complexes was altered. In endothelial IGF-1R knockdown eWAT, there was no significant difference in complex I-mediated leak respiration rates or ADP-linked complex I-mediated respiration, demonstrating no difference in NADH-linked substrate processes. However, upon the addition of succinate at a saturating level to activate complex II, a higher rate of complex I- and II-mediated respiration occurred suggesting that mitochondria from eWAT from mice with endothelial IGF-1R knockdown were better equipped to utilise complex I and II substrates. The higher complex I- and II-mediated respiration suggests that the resultant increased ATP synthesis capacity in eWAT mitochondria from mice with endothelial IGF-1R knockdown may enable more efficient beneficial AT remodelling^65,66^. As with the remodelling changes, we noted these changes were predominantly eWAT specific and not seen in other AT depots. As visceral WAT is most associated with metabolic diseases, a depot-specific effect to improve metabolism, function and phenotype in this depot has potential therapeutic applications.

To probe the mechanisms underpinning these favourable changes, we investigated the possibility that adipocytes were directly derived from IGF-1R knockdown endothelial cells, as it has previously been demonstrated that adipocytes of endothelial origin exist^35^. Using lineage tracing, we did not see this in our model. Since studies continue to emerge supporting the endothelium as a paracrine organ^25–28^, we therefore, investigated a potential paracrine mechanism facilitating cross-talk between the endothelium and eWAT. Treatment of primary adipocytes with conditioned media from primary IGF-1R knockdown endothelial cells after two weeks HFD, even after protein denaturation, led to significant changes in gene expression in human primary adipocytes. A metabolomics approach demonstrated a significant difference in the small molecule endothelial secretome, demonstrating that the secretome of endothelial small molecules may contribute to the pathophysiology of obesity.

A screen of the upregulated metabolites released from IGF-1R knockdown endothelial cells revealed that malonic acid was sufficient to upregulate *Ucp1*, and several other genes involved in adipocyte function. This is in keeping with the recent finding by Oki and co-workers, that malonate can induce browning of WAT^67^. We, therefore, began to probe the effects of malonate in the setting of obesity and its associated metabolic complications. To do this, we employed a malonate prodrug^46^, to enhance cell and tissue uptake, and found, in vitro that MAM could upregulate VEGFA gene expression and basal respiration in human primary adipocytes. We then explored the therapeutic potential in vivo. We directly injected MAM into eWAT of obese wildtype mice finding that this led to increased expression of genes involved in a range of AT functions, including vascularisation, lipid storage, adipokine production, mitochondria biogenesis, adipocyte differentiation and lipogenesis. This demonstrates therapeutic promise for malonate prodrugs in the context of obesity and its associated metabolic complications.

In conclusion, our data reveal a previously unrecognised non-cell autonomous paracrine mechanism by which a reduction in endothelial cell IGF-1R stimulates beneficial, depot-specific remodelling of WAT in mice challenged with a HFD. Additionally, we find that malonate is released by the endothelium when IGF-1R levels are reduced, functioning as a WAT modulator with therapeutic potential in the context of obesity and related metabolic complications. While the effects on AT in our model are predominantly observed in eWAT, with no demonstrable impact on iWAT—indicating a depot-specific effect—we also observe intriguing changes in BAT. Further research is necessary to understand the potential benefits of endothelial IGF-1R knockdown or malonic acid on the phenotype of BAT.

## Methods

### In vivo animal studies

Experiments approved by the UK Home Office Licence P144DD0D6, PP5104353, PP2103311. Mice with tamoxifen-inducible endothelial cell specific knockdown of the IGF-1R receptor (ECIGF-1R^KD^) and their *lox/lox* control littermates, were bred in house from founder animals (VE-Cre #MGI 3848984, Igf1r^(lox)^ #MGI:J:60711^68^, mTmG #MGI:J:124702^69^). Mice were group housed in cages of up to five animals. Only male mice were used for experimental procedures to prevent variability associated with the oestrous cycle on adiposity and metabolic readouts^70,71^. Cages were maintained in humidity- and temperature-controlled conditions (humidity 55% at 22 °C) with a 12 h light-dark cycle and on standard chow diet unless stated (SDS #CRM (P)). Genotyping was carried out by Transnetyx commercial genotyping using ear biopsies’. At 5 weeks old, mice were injected with tamoxifen (T5648 Sigma, dissolved in, Corn Oil—also from Sigma, C8267) (1 mg/day intra-peritoneal for 5 consecutive days). To induce obesity, 8 week old male mice received high fat diet *ad libitum* for either 2 weeks or 8 weeks as stated in figures (60% of energy from fat) (F3282, Bioserve) with the following composition: protein 20.5%, fat 36% and carbohydrate 36.2% (5.51 kcal/g).

### Genotyping

VE-cre reaction mix; 0.5 μl 10 μM Forward Primer: 5′-GCATTACCGGTCGATGCAACGAGTGATGAG-3′ 0.5 μl 10 μM Reverse Primer: 5′-GAGTGAACGAACCTGGTCGAAATCAGTGCG-3′ 10 μl x2 Bio mix red PCR Master Mix, 13 μl water and 1 μl extracted DNA. PCR cycle as follows; Initial denaturation 95 °C for 1 min, denaturation 95 °C for 15 s, annealing 51 °C for 30 s, extension 72 °C for 1 min and final extension 72 °C for 6 min. Denaturation, annealing and extension repeated for 35 cycles. PCR products were then run on a 1.5% agarose gel for 1 h at 110 V, with a 100 bp ladder. Expected product sizes are Cre Positive—408 bp.

IGF-1R lox reaction mix; 1 μl 10 μM Forward Primer1: 5′-CTTCCCAGCTTGCTACTCTAG G-3′ 1 μl 10 μM Forward Primer2: 5′-TGAGACGTAGCGAGATTGCTGTA-3′ 1 μl 10 μM Reverse Primer: 5′-CAGGCTTGCAATGAGACATGGG-3′ 10 μl x2 Bio mix red PCR MasterMix, 11 μl water and 1 μl extracted DNA. PCR cycle as follows; Initial denaturation 94 °C for 4 min, denaturation 94 °C for 45 s, annealing 61 °C for 45 s, extension 72 °C for 1 min and final extension 72 °C for 5 min. Denaturation, annealing and extension repeated for 35 cycles. PCR products were then run on a 1.5% agarose gel for 1 h at 110 V, with a 100 bp ladder. Expected products sizes are; Control–120 bp, Homozygous–220 bp and Heterozygous–120 & 220 bp.

mTmG reaction mix; 0.5 μl 10 μM Common Primer: 5′- CTCTGCTGCCTCCTGGCTTCT-3′ 0.5 μl 10 μM Control Reverse Primer: 5′-CGAGGCGGATCACAAGCAATA-3′ 0.5 μl 10 μM Mutant Reverse Primer: 5′-TCAATGGGCGGGGGTCGTT-3′ 10 μl x2 Bio mix red PCR Master Mix, 12.5 μl water and 1 μl extracted DNA. PCR cycle as follows; Initial denaturation 94 °C for 2 min, denaturation 94 °C for 30 s, annealing 62 °C for 30 s, extension 72 °C for 30 s and final extension 72 °C for 10 min. Denaturation, annealing and extension repeated for 35 cycles. PCR products were then run on a 1.5% agarose gel for 1 h at 110 V, with a 100 bp ladder. Expected product sizes are; Control–330 bp, Homozygous–250 bp, and Heterozygous–250 and 350 bp.

### Confirmation of tamoxifen induction of mT to mG

Founder mTmG mice were obtained from the Jackson Laboratory (Bar Harbor, ME, USA). In the absence of Cre recombinase, mTmG mice constitutively express mTdTomato, a non-oligomerising DsRed variant. After tamoxifen induction and therefore following exposure to Cre recombinase and excision of the mTdTomato expression cassette, the rearranged mTmG transgene converts to the expression of mGFP (green fluorescent protein). Both mTdTomato and mGFP are membrane-targeted, allowing for delineation of single cells using fluorescence microscopy. Mice were perfuse-fixed with 4% paraformaldehyde (PFA). Femoral arteries were excised, permeabilised (0.1% TritonX-100 in PBS) and blocked (Serum free protein block, DAKO), before overnight incubation with a rabbit polyclonal antibody to mouse CD31 (ab28364, Abcam) followed by overnight incubation with a goat polyclonal anti-rabbit conjugated to Chromeo642 (ab60319, Abcam, UK). Arteries were then mounted *en face* on slides using DAPI (DAPI-Fluoromount-G, Southern Biotech) to define nuclei. Confocal microscopy (LSM 700, Zeiss, UK) was used to define CD31, mGFP and mTdTomato fluorescence.

### Glucose and insulin tolerance testing

Mice were fasted overnight prior to glucose tolerance tests or for 2 h prior to insulin tolerance tests. Blood glucose was measured using a handheld Glucose Metre (Accu-Chek Aviva). An intra-peritoneal injection of glucose (1 mg/g) *or recombinant* human insulin (Actrapid; Novo Nordisk) (0.75 IU/kg) was given and glucose concentration measured at 30 min intervals for 2 h from the point of glucose/insulin administration. Mice were not restrained between measurements^72,73^. Data were analysed using GraphPad Prism Area under the curve (AUC) calculations.

### Metabolic phenotyping

Metabolic parameters were measured by indirect calorimetry using Comprehensive Lab Animal Monitoring Systems (CLAMS)(Columbus Instruments). In brief, mice were individually housed for 5 days and measurement of their oxygen consumption, carbon dioxide production, food intake, and locomotor activity were continuously recorded^74^. For each mouse, a full 24 h period, taking into account sleep and wake cycles, was analysed after an acclimatisation period^75^. Core body temperature was measured using a rectal temperature probe (Vevo2100 (Visualsonics, FujuFilm) with an Indus rectal temperature probe)^28^.

### Plasma samples

Fasting plasma samples were collected from the lateral saphenous vein (EDTA collection tubes Sarstedt 16.444) and spun at 2000 × *g* for 10 min in a bench top centrifuge. Fasting plasma insulin (90080, CrystalChem), IGF-I (MG100, R and D systems), leptin (EZML-82K, Merk-Millipore), adiponectin (EZMADP-60K, Merk-Millipore), triglycerides (Abcam Ab65336) and free fatty acids (Abcam, ab65341) were measured as per manufactures instructions.

### Murine tissue samples

After either 2 or 8 weeks of high fat feeding, all mice were sacrificed using terminal anaesthesia using 5% isoflurane, followed by terminal blood collection taken via cardiac puncture followed by organ removal. Organ weights were measured using a standard laboratory balance.

### Quantification of gene expression

RNA was isolated from tissue and cells samples (NEB, T2010S). The concentration of RNA in each sample (ng/ul) was measured using a Nanodrop. cDNA was reverse transcribed from the RNA samples (NEB, E3010L). Quantitative PCR (qPCR) was performed using a Roche LightCycler 480 Instrument II, using SYBR Green PCR Master Mix (Bio-Rad, 1725120) and relevant primers (Supp Table 1). The ‘cycles to threshold’ (cT) was measured for each well, the average of triplicate readings for each sample taken, normalised to GAPDH or 18 s rRNA, and finally the differential expression of each gene was calculated for each sample^28^.

### Histological assessment

Samples for histology were fixed in 4% PFA for at least 24 h and then processed into paraffin blocks. 5 µm sections were taken, slides were stained with haematoxylin and eosin to assess gross morphology or Picro-sirius red for collagen deposition. Slides were imaged using an Olympus BX41 microscope at 10x and 20x magnification. For assessment of adipocyte size, three separate fields of view for each sample were assessed. For each one, the average was taken of 20 randomly selected independent cells measured using ImageJ. For Macrophage infiltration, shown by CLS, the presence and number of CLS were assessed within the tissue slide^76^. Each sample was assessed by at least two blinded independent verifiers (NJH or KIB), and the average score per sample taken.

For collagen deposition, the percentage of the sample staining positive for collagen using picrosirus red was measured using thresholding in ImageJ, and again was taken as the average in at least three independent areas of the sample.

For assessment of non-alcoholic fatty liver disease (NAFLD) in sections of murine liver, a validated rodent NAFLD scoring system was used^77^, which takes into account micro and macro-steatosis, inflammation and hypertrophy. Each sample was assessed by at least two blinded independent verifiers (NJH, KIB or NW), and the average score per sample taken.

### Quantification of tissue vascularity

AT was fixed in 1% PFA, and allowed to fix for 2 h at room temperature; samples were transferred into phosphate buffered saline (PBS) for longer storage. Samples were incubated overnight with Isolectin B4 Alexa Fluor 647 (I32450, Thermo Fisher Scientific) (for murine samples), diluted 1:100 in 5% BSA in PBS at 4 °C. After washing with PBS, they were incubated with HCS LipidTOX (H34475, Thermo Fisher Scientific) diluted 1:200 in PBS for 20 min at room temperature. Whole tissue sections were then mounted onto slides beneath cover slips using a silicone spacer (Grace bio-labs, 664113), with Prolong Gold (P36930, Thermo Fisher Scientific) and imaged using laser scanning confocal microscopy (LSM880, Zeiss). Vascular density (the proportion of each image stained with lectin) was measured using thresholding in ImageJ. Green staining is a composite of GFP and Lipidtox in mice samples, green is not quantified.

Organs (muscle and liver) were harvested under terminal anaesthesia and fixed in 4% PFA for 1 h at room temperature. Organs were then embedded in Optimal Cutting Temperature compound (OCT) (Cellpath, KMA-0100-00A) and stored at −80 until sectioned. 10 µM sections were taken using a Leica CM3050 S Research Cryostat. Slides were blocked and permeabilised in PBS + 0.25% Triton-X100 + 1% BSA + for one hour, then stained with Isolectin B4-Alexa Fluor-488 (Invitrogen I21411) at 1/100 in PBS + 0.25% Triton + 1% BSA for 1 h. Slides were washed three times in PBS and mounted with a coverslip using Prolong Gold with DAPI (P36931, ThermoFisher). Slides were then imaged using laser scanning confocal microscopy (LSM880, Zeiss), with 8 areas of each sample imaged. Vascular density (the proportion of each image stained with IB4) was measured using thresholding in ImageJ.

### Assessment of neovascularisation

Angiogenesis assays from AT were performed using a modified technique^78^. In sterile conditions, any surface blood vessels were dissected from the adipose tissue sample, before it was cut into pieces no bigger than 1 mm^3^. For each sample, at least 20 sections were individually embedded into a fibrin matrix in each well of a 24 well tissue culture plate. The fibrin matrix was achieved by combining 12.5 μl of 50 U/ml thrombin (Sigma-Aldrich T-3399) with 500 μl of a mix containing 4 U/ml aprotinin (Sigma-Aldrich A-1153) and 2 mg/ml fibrinogen type 1 (Sigma-Aldrich F-8630), and adding a piece of adipose tissue into the well before the matrix had set. The plates were then incubated at room temperature for 20 min, and then at 37 °C for a further 20 min to ensure that the matrix had fully formed around the piece of adipose tissue. One ml of media was then carefully pipetted onto the top of each well, and plates were cultured at 37 °C, 5% CO_2_ for up to 7 days. The media was discarded and replaced every other day throughout the culture period. Each day, the samples were imaged at 4x magnification on Olympus florescent microscope CKX41 and number of endothelial sprouts coming from each piece of fat was counted. For each sample, the average number of sprouts per section was calculated, as well as the number of sections which had sprouted.

### High-resolution tissue respirometry in adipose tissue

Mice were sacrificed using cervical dislocation. Subcutaneous inguinal WAT (iWAT) and visceral epididymal WAT (eWAT) were placed into biopsy preservation solution for high-resolution respirometry (BIOPS)^74,79^. Tissue was permeabilised with freshly prepared 5 μg/mL digitonin (Cayman Chemical #14952) and washed in mitochondrial respiration medium, MiR05 (MiR05: 110 mM sucrose, 60 mM K-lactobionate, 20 mM HEPES, 20 mM taurine, 10 mM KH2PO4, 3 mM MgCl2, 0.5 mM EGTA, 0.1% (w/v) fatty acid-free BSA, pH 7.1).

Using OxygraphO2k high resolution respirometry (OROBOROS) we measured respiration in tissue slices of the adipose depots^74,79^. Substrates were added to the chamber in the subsequent order. Final concentrations in the chamber provide in brackets. Complex I-mediated respiration was measured with glutamate (10 mM), malate (1 mM) and ADP (2.5 mM). Complex I and II supported oxidative phosphorylation was measured with Succinate (10 mM). Respiration rates not contributed to by UCP1 were measured by Guanosine 5′-diphosphate sodium salt (GDP) which was added at 1 mM at a time. Maximal ETC capacity was measured by carbonyl cyanide m-chlorophenyl hydrazine (CCCP; 5 μM in DMSO). C complex II mediated oxidative phosphorylation was measured by Rotenone (0.5 μM). Nonmitochondrial (background) respiration was measured by Antimycin A (12.5 μM). This background was subtracted as a correcting factor from all respiratory measurements. The stabilised slope of the oxygen flux was calculated as the rate of respiration. Data was collected and analysed on Datlab software version 6.1 (Oroboros Instruments, Austria)^74,79^.

### Lipolysis

Basal and adrenergic stimulated lipolysis were measured using an adapted published protocol^80^. Briefly, eWAT was transferred into pre-warmed Krebs-Ringer Bicarbonate HEPES (KRBH) buffer (without 2% bovine serum albumin [BSA]), pH 7.4. The tissue was then transferred to a sterile 15 cm tissue culture dish. Using sterile scissors, 50 mg (~0.5 mm³) of the adipose tissue was cut and placed into individual wells of a 24-well plate containing 1 mL of pre-warmed KRBH buffer supplemented with 2% fatty acid-free BSA (KRBH-BSA buffer). After the tissue was plated, the medium from each well was aspirated and replaced with KRBH-BSA buffer containing either a vehicle (control) or 10 μM forskolin (Merck #F6886). Five minutes post-treatment, 100 μL of the medium was collected from each well as the baseline sample. Each well was then replenished with 100 μL of fresh medium containing the respective treatment. The tissue was incubated at 37 °C for 4 h, after which 1 mL of medium was collected from each well. Both baseline and 4-h media aliquots were incubated at 65 °C for 10 min to inactivate residual enzymatic activity. After incubation, the tissue samples in each well were washed with 1 mL of phosphate-buffered saline (PBS), and the tissue was stored at −80 °C for subsequent protein quantification. The level of glycerol from all the experimental media was detected and quantified using the Glycerol Assay Kit (Sigma-Aldrich #MAK117) according to the manufacturer’s protocol. Data was normalised to protein content. Lipolytic capacity was calculated as previously reported^33^.

### Quantification of protein expression

Cells were lysed or tissue mechanically homogenised in lysis buffer (Extraction buffer, FNN0011) and protein content was quantified by a BCA assay (Sigma-Aldrich, St. Louis, MO). Twenty micrograms of protein were resolved on a 4–12% Bis-Tris gel (Bio-Rad, Hertfordshire, UK) and transferred to nitrocellulose membranes. Membranes were probed with antibodies diluted 1:1000 in 5% BSA (IGF-1R Cell signalling #9750, p473 AKT Cell Signalling #4060S, and AKT Cell Signalling #9272S), before incubation with appropriate secondary horseradish peroxidase-conjugated antibody. Blots were visualised with Immobilon Western Chemiluminescence HRP Substrate (Merck Millipore, Hertfordshire, UK) and imaged with Syngene chemiluminescence imaging system (SynGene, Cambridge, UK). Densitometry was performed in ImageJ^81^.

### Primary endothelial cell isolation and culture

Primary endothelial cells (PECs) were isolated from lungs^82,83^. Briefly, lungs were harvested, washed, finely minced, and digested in Hanks’ balanced salt solution containing 0.18 units/mL collagenase (1 mg/mL; Roche) for 45 min at 37 °C. The digested tissue was filtered through a 70-μm cell strainer and centrifuged at 300 × *g* for 10 min. The cell pellet was washed with PBS/0.5% BSA, centrifuged, re-suspended in 1 mL PBS/0.5% BSA, and incubated with 1 × 10^6^ CD146 antibody–coated beads (Miltenyi Biotech, 130-092-007) at 4 °C for 30 min. Bead-bound PEC were separated from non–bead-bound cells using a magnet. Cells were re-suspended in 2 ml supplemented endothelial growth medium–MV2 (PromoCell) and seeded on a 6 well fibronectin coated plates. Cells were cultured at 37 °C in 5% CO_2_ with twice-weekly media changes until confluent^81^.

### Conditioned media experiments

When PECs reached confluency, supplemented growth media was removed and replaced with basal endothelial growth medium–MV2 (Promocell) for 24 h. Conditioned media was then removed and used in further experiments as described.

Primary human subcutaneous white preadipocytes (Lonza # PT-5020; human male donor, BMI of 30, age 60 years, Caucasian) were seeded (10,000 cells/cm^2^) in 24 well plates (Costar, Corning, NY, USA) and grown until confluent (37 °C, 5% CO_2_) in PromoCell Preadipocyte Growth Medium (C-27410, 0.05 mL/mL foetal calf serum, 0.004 mL/mL endothelial cell growth supplement, 10 ng/mL epidermal growth factor, 1 µg/mL hydrocortisone, 90 µg/mL heparin)^74^. To differentiate confluent pre-adipocytes, growth medium was replaced by PromoCell Adipocyte Differentiation Medium (C-27436, 8 µg/mL d-Biotin, 0.5 µg/mL insulin, 400 ng/mL dexamethasone, 44 µg/mL IBMX, 9 ng/mL L-thyroxine, 3 µg/ml ciglitazone) for 48 h (day 0). Differentiation medium was subsequently replaced (day 2) with PromoCell Adipocyte Nutrition Medium (C-27438, 0.03 mL/mL foetal calf serum, 8 µg/mL d-Biotin, 0.5 µg/mL insulin, 400 ng/mL dexamethasone) for the remainder of the differentiation period (up to day 14). All Cell medium was supplemented with 1% penicillin-streptomycin (10,000 units/mL penicillin, 10 mg/mL streptomycin). Conditioned media was then added to the differentiated human adipocytes for 24 h before the cells were lysed and RNA extracted for gene expression analysis.

### Separation of conditioned media into aqueous and lipid fractions

600 µL of 2:1 methanol:chloroform was added to 1 mL of conditioned media, followed by 200 µl of water and an additional 200 µL of chloroform, vortexed and then centrifuged at 13100 *g* for 20 min. The top layer (aqueous layer containing the aqueous metabolites) was pipetted off and placed into the evacuation centrifuge at 40 °C for 6 h. The protein disc (middle layer) was discarded and the final bottom layer (containing lipid metabolites) was transferred into a clean Eppendorf and left overnight in a fume hood at room temperature until all chloroform had evaporated. Both the lipid and aqueous metabolites were stored at −80 °C.

### Aqueous sample preparation

Samples were reconstituted in 1 mL sample resuspension buffer (95% acetonitrile and 5 % mobile phase A). Mobile phase A = 95% water, 5% acetonitrile, 20 mM ammonium acetate and 20 mM ammonium hydroxide, pH = 9. Samples were vortex mixed and the extracted metabolites were transferred to a 2 mL glass vial.

### Liquid chromatography

A SCIEX ExionLC™ AD HPLC system with a Luna 3 µm NH2 100 Å, 150 × 4.6 mm column (Phenomenex) was used. Mobile phase A = 95% water, 5% acetonitrile, 20 mM ammonium acetate and 20 mM ammonium hydroxide, pH = 9; Mobile phase B = 95% acetonitrile and 5% mobile phase A and 20 mM ammonium hydroxide. The flow rate was set at 350 µL/min. The wash solvent for the autosampler was 20/20/60 methanol/acetonitrile/isopropanol. The injection volume was 2 µL, and the column was kept at 40 °C. The gradient method was 100% B for 2 min, then to 85% B for 3 min, then to 30% for 10 min, then to 2% B for 5 min, then 100% for 10 min. *n* = 4 per group.

### Mass spectrometry

A SCIEX QTRAP® 6500+ with IonDrive Turbo V source was used. MS source parameters are Curtain Gas was 30 psi for both (+) and (-). Collision Gas was high for both (+) and (-), Ionspray voltage was 5500 V for (+) and −4500 V for (-). Temperature was 500 °C for both (+) and (-), Ion source gas 1 was 35 psi for both (+) and (-), ion source gas was 45 psi for both (+) and (-), delustreing potential was 93 V for (+) and −93 V for (-), entrance potential was 10 V for (+) and −10 V for (-) and collision cell exit potential was 10 V for (+) and −10V for (-). *n* = 4 per group.

### Metabolite screening

Mouse 3T3-L1 preadipocytes (ATCC #CL-173) were cultured in 10% (v/v) CS/DMEM containing 4.5 g/l glucose and 1 mM Sodium Pyruvate and supplemented with 1XAntibiotic Antimycotic Solution and incubated at 37 °C in 5% CO_2_ for two days upon splitting. Two days after splitting, the media was replaced by 10% (v/v) FBS/DMEM to grow the cells to confluency. After two days of post-confluency (equivalence of day 0), adipocyte differentiation was initiated with MDI induction media (10% FBS/DMEM, 0.5 mM IBMX, 1 µM dexamethasone and 1 µg/mL insulin). On day 2, the MDI induction media was replaced by insulin media (10% FBS/DMEM supplemented with 1 µg/mL insulin). From day 4 onwards, the media was replaced by 10% FBS/DMEM every two days. Full differentiation was achieved between day 7 and day 10. Mature adipocytes were subjected to metabolite stimulation. Metabolites, Malonic acid^84^ or pro-drugs^46^ (Table 1) and their solvents, DPBS or DMSO, were applied for 24 h at 37 °C in 5% CO_2_.

**Table 1:** Metabolite details

Metabolite/prodrug	Final Conc	Manufacturer	Cat #
2′-deoxycytidine triphosphate (dCTP)	10 mM	Thermo Scientific	R0151
2′-deoxyuridinemonophosphate	2 mM	Santa Cruz	sc-214058
Arginine	2 mM	Alfa Aesar	11498850
Citric acid specific	2 mM	SIGMA-ALDRICH	C0759
Cytidine monophosphate (CMP)	2 mM	Fluorochem	47062
Folic acid	11.3 μM	SIGMA-ALDRICH	F7876
Glucosamine 6 P (G6P)	2 mM	Santa Cruz	sc-214809
Glucuronic acid	10 ng/ml	SIGMA-ALDRICH	G5269
Glutamine	2 mM	Thermo Fisher	25030032
Guanosine diphosphate (GDP)	10 μM	MedChemExpress	HY113066A
Guanosine-3′5-‘cyclic monophosphate	200 μM	SIGMA-ALDRICH	G7504
Inosine	100 μM	Alfa Aesar	A14459.06
Inosine monophosphate (IMP)	1.25 mM	SIGMA-ALDRICH	57510
Leucine	2 mM	G BIOSCIENCES	RC-064
Maleic acid	8 mM	Acros Organics	10396760
Malonic acid	10 mM	Alfa Aesar	11464523
NADP reduced (NADPH)	2 mM	Santa Cruz	sc-202725
Pantothenic acid	1 mM	SIGMA-ALDRICH	21210
Phthalic acid	10 μM	SIGMA-ALDRICH	P39303
Proline	15 nM	Alfa Aesar	A10199.14
Quinolinic acid	5 mM	SIGMA-ALDRICH	160660
Riboflavin	1 μM	Alfa Aesar	A11764
S-5-Adenosyl-L-Cysteine	100 μM	SIGMA-ALDRICH	A7772
Shikimic acid	80 μM	Acros Organics	10533491
Thymidine diphosphate (TDP)	2 mM	SIGMA-ALDRICH	T9375
Uridine diphosphate (UDP)	2 mM	SIGMA-ALDRICH	U4125
Phosphatidylglycerol 18:0 (PG (18:0))	2 mM	SIGMA-ALDRICH	840465 P
alpha-linolenic acid	300 μM	Santa Cruz	sc-205545

### Adiponectin secretion

After stimulation of the mature 3T3-L1 adipocytes, the conditioned media was collected and centrifuged at 2000 × *g* for 10 min at 4 °C to pellet cell debris. The supernatants were then used to quantify the level of adiponectin using Mouse Adiponectin ELISA kit (Merck Millipore #EZMADP-60K) according to the manufacturer’s instructions.

### Mitochondria-targeted antioxidant

100 nM Mitoquinone (MitoQ; MedChemExpress LLC #HY-100116A) was applied 30 min prior to 24-h malonic acid treatment^45^.

### FGFR1 blocker

20 nM PD173074 (Apexbio #A8253) was applied an hour prior to 24-h malonic acid treatment^85^.

### High-throughput respirometry of human adipocytes

To measure mitochondrial respiration, stimulated human white adipocytes were subjected to Seahorse XF Cell Mito Stress Test using Seahorse XF Cell Mito Stress Test Kit (Agilent #103015-100) on a Seahorse XFe96 Analyzer (Agilent Technologies) according to the manufacturer’s instructions.

At the end of the prodrug stimulation, cells were washed with and then incubated with Seahorse Mito Stress Test Assay Medium (Seahorse XF Base Medium, supplemented with 10 mM glucose, 1 mM sodium pyruvate and 2 mM glutamine, pH 7.4, Agilent #103680-100) in a non-CO2 incubator at 37 °C for 45–60 min prior to the assay.

The microplate was then loaded onto a Seahorse XF 96 Analyzer. Oxygen consumption rate (OCR) was measured for quantification of different respiratory parameters throughout the assay. A series of injections were performed: 1.5 μM oligomycin, 1 μM carbonyl cyanide-p- trifluoromethoxyphenyl-hydrazon (FCCP) and 0.5 μM rotenone in conjunction with 0.5 μM antimycin A. To inhibit the activity of mitochondrial ATP synthase, oligomycin was injected first. To elicit maximal mitochondrial respiration by disrupting the proton gradient, FCCP was then injected. Finally, to suppress the activity of mitochondrial complex I and III respectively, rotenone and antimycin A (Rot/AA) were injected. Data was normalised to nuclear DNA content which was determined using fluorescence staining (ThermoFisher #H1399). Fluorescence was measured by Cytation 5 Cell Imaging Multimode Reader (BioTek) (excitation at 350 nm; emission at 461 nm).

### Glucose uptake assay

To evaluate the effect of malonic acid on glucose uptake, mature 3T3-L1 adipocytes were cultured and treated with 10 mM malonic acid or DPBS in supplement-free MV media. At the end of the stimulation, adipocytes were washed in DPBS and then incubated in low-glucose (1 g/L) serum-free DMEM medium (VWR #392-0407) for 1 h at 37 °C and 5% CO_2_^74^. 30 min before the end of the 1-h incubation, adipocytes were treated with 300 μg/mL 2-NBDG (ThermoFisher #N13195). At the end of the incubations, adipocytes were washed with DPBS three times. The plate was then subjected to fluorescence reading using CLARIOstar multi-mode microplate reader (BMG LABTECH, Germany) (excitation at 483 nm; emission at 530 nm). Data was normalised to nuclear DNA content which was determined using Hoechst staining. Cells were incubated with 2 μg/mL Hoechst 33342 staining solution (ThermoFisher #H1399) for 20 min at room temperature. To remove excess stain, the cells were washed with DPBS three times. After the last wash, fluorescence was measured by CLARIOstar multi-mode microplate reader (BMG LABTECH, Germany) (excitation at 355 nm; emission at 455 nm).

### In vivo MAM treatment

C57BL/6 mice at 6 weeks old were purchased from Charles River Laboratories. At 8 weeks old mice received 60% HFD (Bio-Serv #F3282) for 8 weeks. A single dose of MAM or the vehicle control (0.9% saline and 0.1% DMSO) was injected into the left epididymal white adipose pad. The mice were allowed to recover for 24 h with free access to food (60% HFD) and water. Then, mice were sacrificed by cervical dislocation. Epididymal white adipose tissue was excised and snap-frozen in liquid nitrogen.

### Quantification and statistical analysis

Priori sample size calculations for animal experiments were performed using our published pilot data using the online software package from Vanderbilt University for multiple types of power analysis (https://biostat.app.vumc.org/wiki/Main/PowerSampleSize). All data are shown as mean ± SEM. Individual mice or replicates are shown as individual data points. All image analysis was performed in ImageJ. Student 2-tailed unpaired t-test or one-way ANOVA (where appropriate) were used for statistical analyses and were performed with GraphPad Prism software version 7. * denotes *P* ≤ 0.05 and ** *P* ≤ 0.01. Exact details can be found in figure legends.

### Reporting summary

Further information on research design is available in the Nature Portfolio Reporting Summary linked to this article.

## Supplementary information

Supplementary Information

Reporting Summary

## Source data

Source Data

## Data Availability

All the data supporting the findings described in this manuscript are available in the article and in the Supplementary Information. Source data are provided with this paper.
