# Supplementary material for: Paracrine role of endothelial IGF-1 receptor in depot-specific adipose tissue adaptation in male mice
Source: Nat Commun. 2025 Jan 2;16:170. doi: 10.1038/s41467-024-54669-1 (PMC11696296; doi:10.1038/s41467-024-54669-1)
Supplement: Supplementary file 1 — Supplementary Information [file 41467_2024_54669_MOESM1_ESM.pdf]

Supplementary Figure 1

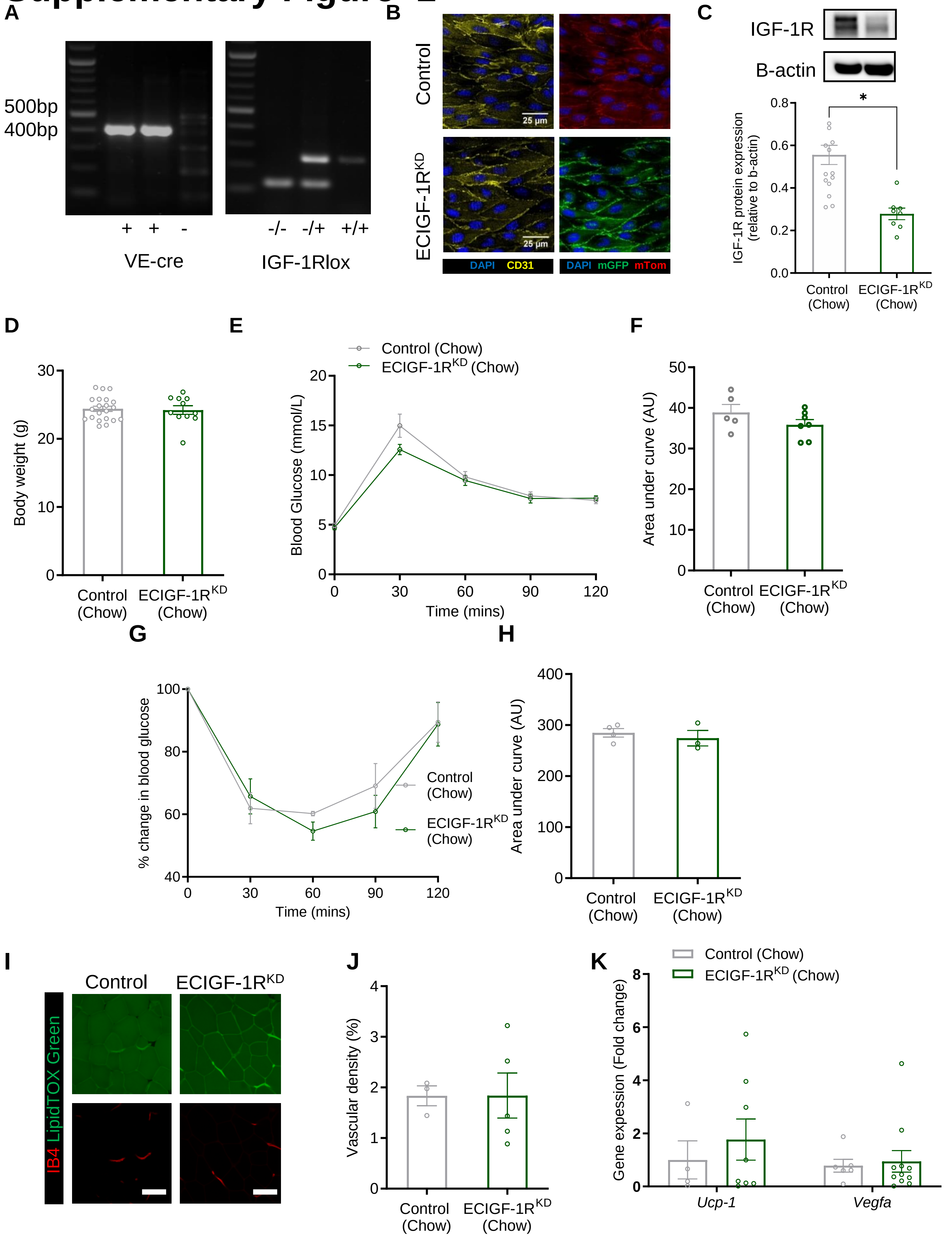

Supplementary figure 1 – Confirmation of IGF-1R reduction in murine model of endothelial specific IGF-1R knockdown

A. Representative images of genotyping during the generation of tamoxifen-inducible endothelial cell specific IGF-1R knockdown mice (ECIGF-1R^{KD}) mice.

B. Representative images of *en face* stained femoral arteries from tamoxifen-induced ECIGF-1R^{KD} mice and control littermates confirming a switch from mT to mG with tamoxifen (Scale bar = 25µm).

C. Quantification of primary murine endothelial cell expression of IGF-1R from chow fed control and ECIGF-1R^{KD} mice (n =16&8).

D. Quantification of body weight from chow fat fed control and ECIGF-1R^{KD} mice. (n=11&20).

E. Glucose tolerance over time chow fed control and ECIGF-1R^{KD} mice (n=5&7).

F. Area under the curve (AUC) analysis for glucose tolerance for chow fed control and ECIGF-1R^{KD} mice (n =5&7).

G. Insulin tolerance test for chow fed control and ECIGF-1R^{KD} mice (n =4&3).

H. The area under the curve analysis for insulin tolerance tests for chow fed control and ECIGF-1R^{KD} mice (n =4&3).

I. Representative images of isolectin B4 (Red) and LipidTox (Green) stained white epididymal adipose tissue (eWAT) from chow fed control and ECIGF-1R^{KD} mice (Scale bar = 100µm).

J. Quantification of vascularity in eWAT from chow fed control and ECIGF-1R^{KD} mice (n =3&5).

K. Quantification of *Ucp-1* and *Vegfa* gene expression in white epididymal adipose tissue from chow fed control and ECIGF-1R^{KD} mice (n =4-11).

Data shown as mean ± SEM, data points are individual mice. $p<0.05$ taken as statistically significant using student unpaired two tailed t-test and denoted as *.

Supplementary Figure 2

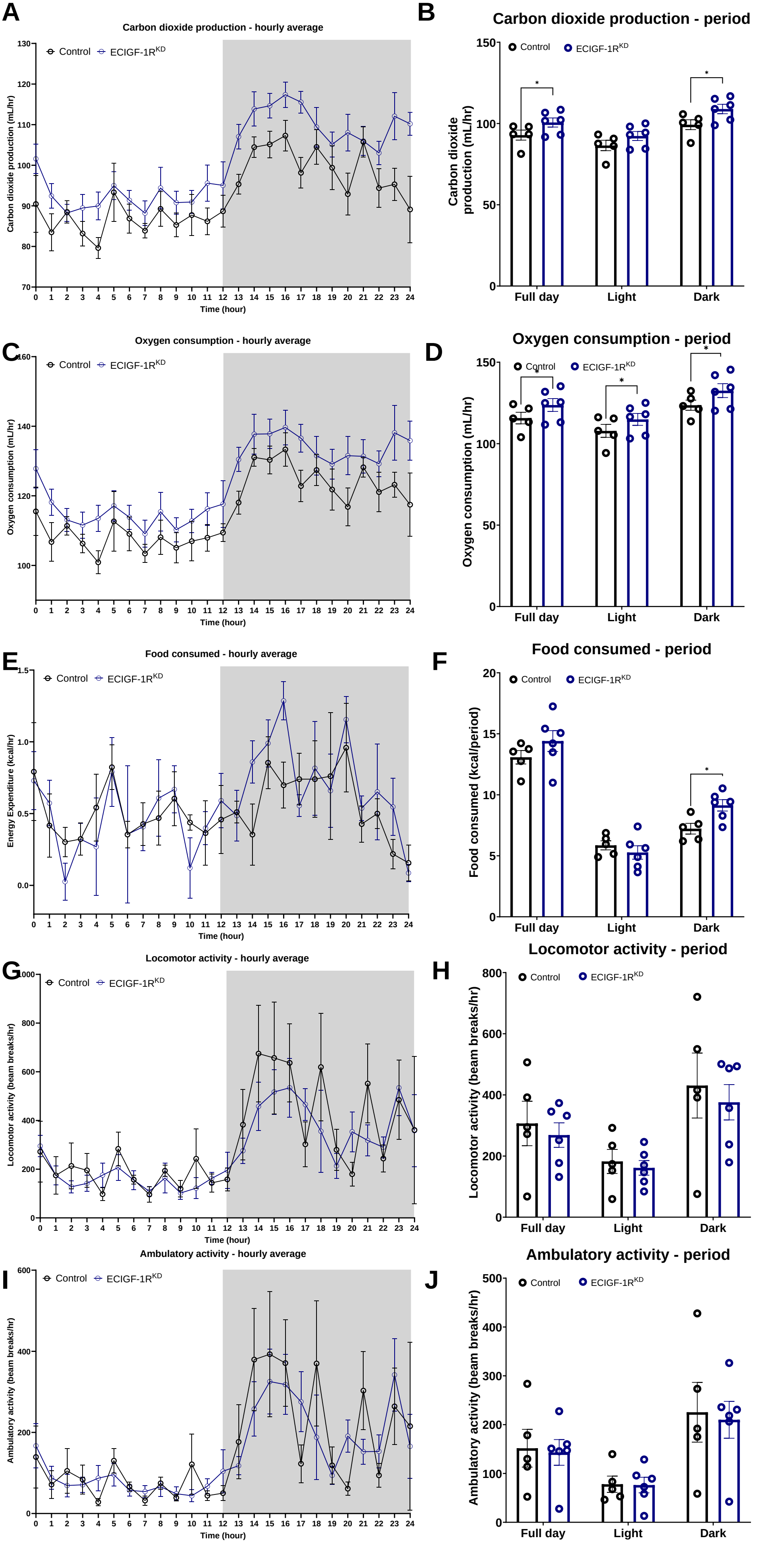

Supplementary figure 2 – Characterisation of energy expenditure in mice with reduced endothelial IGF-1R expression after 2 weeks of high fat diet

A. Carbon dioxide production for 2-week high fat diet (HFD) control and tamoxifen-inducible endothelial cell specific IGF-1R knockdown mice (ECIGF-1R^{KD}) mice over 24 hour period (n= 5&6).

B. Average carbon dioxide production for 2-week HFD control and ECIGF-1R^{KD} mice (n= 5&6).

C. Oxygen consumption for 2-week HFD control and ECIGF-1R^{KD} mice over 24 hour period (n= 5&6).

D. Average oxygen consumption for 2-week HFD control and ECIGF-1R^{KD} mice (n= 5&6).

E. Food consumption for 2-week HFD control and ECIGF-1R^{KD} mice over 24 hour period (n= 5&6).

F. Average food consumption for 2-week HFD control and ECIGF-1R^{KD} mice (n= 5&6).

G. Locomotor activity levels for 2-week HFD control and ECIGF-1R^{KD} mice over 24 hour period (n= 5&6).

H. Average locomotor activity levels for 2-week HFD control and ECIGF-1R^{KD} mice (n= 5&6).

I. Ambulatory activity levels for 2-week HFD control and ECIGF-1R^{KD} mice over 24 hour period (n= 5&6).

J. Average ambulatory activity levels for 2-week HFD control and ECIGF-1R^{KD} mice (n= 5&6).

The light/dark cycle for graphs A,C,E,G are shown as follows; Light in white and dark in grey. Data shown as mean ± SEM, $p<0.05$ taken as being statistically significant using student t-test and denoted as *. Metabolic parameters were measured by indirect calorimetry, ANOVA testing was performed using calrapp.org.

Supplementary Figure 3

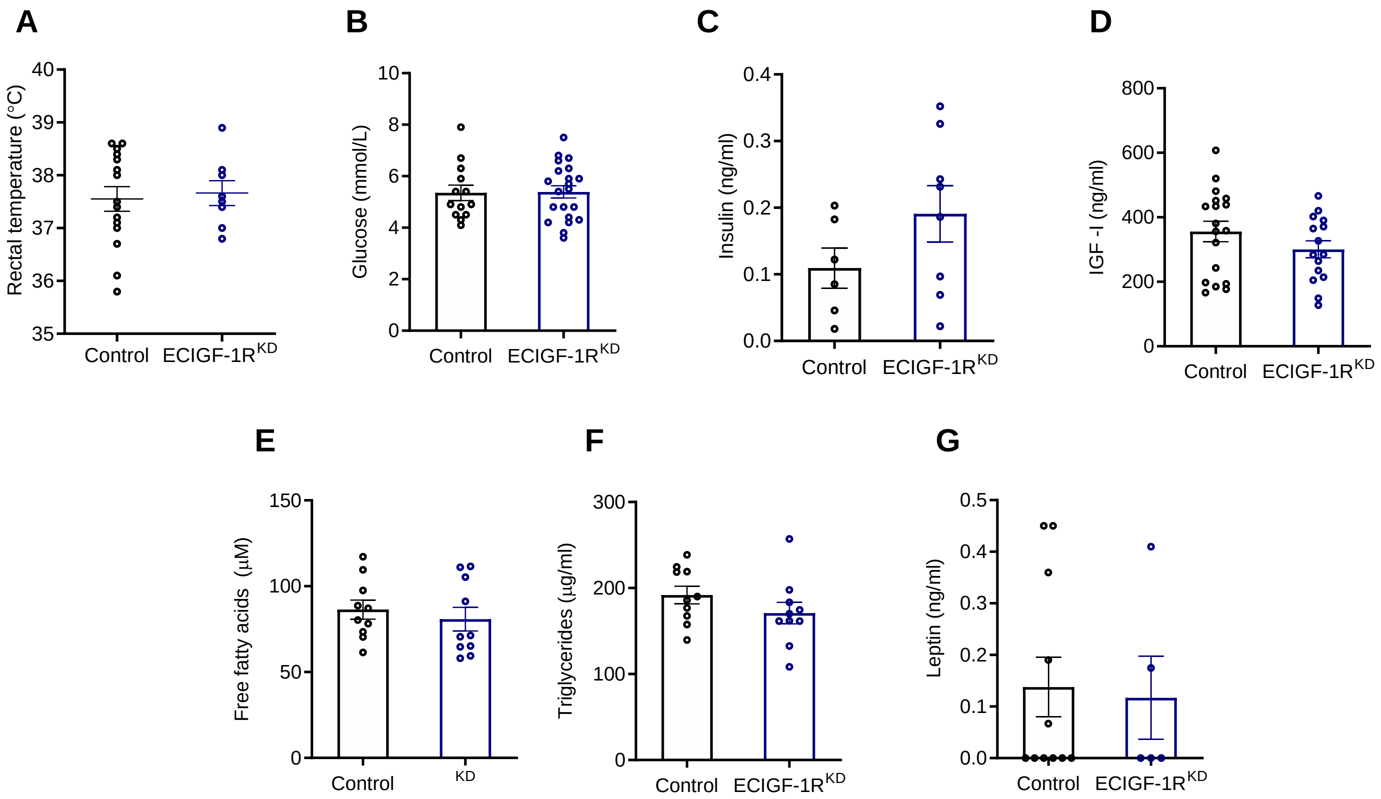

Supplementary figure 3 – No difference in metabolic plasma markers from mice with endothelial specific IGF-1R reduction in the setting of over nutrition

A. Core body temperature of 2-week high fat diet (HFD) control and tamoxifen-inducible endothelial cell specific IGF-1R knockdown mice (ECIGF-$1R^{KD}$) mice (n =15&8).

B. Fasting blood glucose levels of 2-week HFD control and ECIGF-$1R^{KD}$ mice (n =13&21).

C. Fasting plasma insulin levels from 2-week HFD control and ECIGF-$1R^{KD}$ mice (n =6&8).

D. Fasting circulating plasma IGF-I levels from 2-week HFD control and ECIGF-$1R^{KD}$ mice (n =18&15).

E. Fasting plasma free fatty acids levels from 2-week HFD control and ECIGF-$1R^{KD}$ mice (n =10&10).

F. Fasting plasma triglyceride levels from 2-week HFD control and ECIGF-$1R^{KD}$ mice (n =10&10).

G. Fasting plasma leptin levels of 2-week HFD control and ECIGF-$1R^{KD}$ mice (n =11&5).

Data shown as mean ± SEM, data points are individual mice. $p<0.05$ taken as being statistically significant using student unpaired two tailed t-test and denoted as *.

Supplementary Figure 4

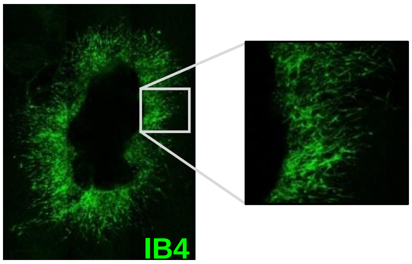

Supplementary figure 4 – Confirming endothelial cells in neovascularisation

Staining of epididymal white adipose tissue explants confirms sprouts are endothelial with positive isolectin B4 staining (green).

Supplementary Figure 5

A

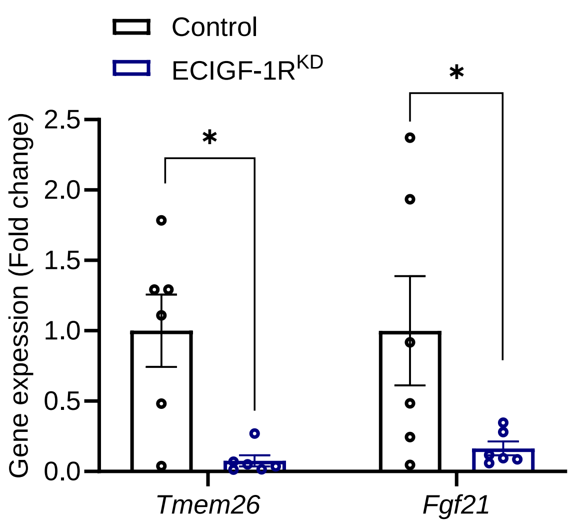

B

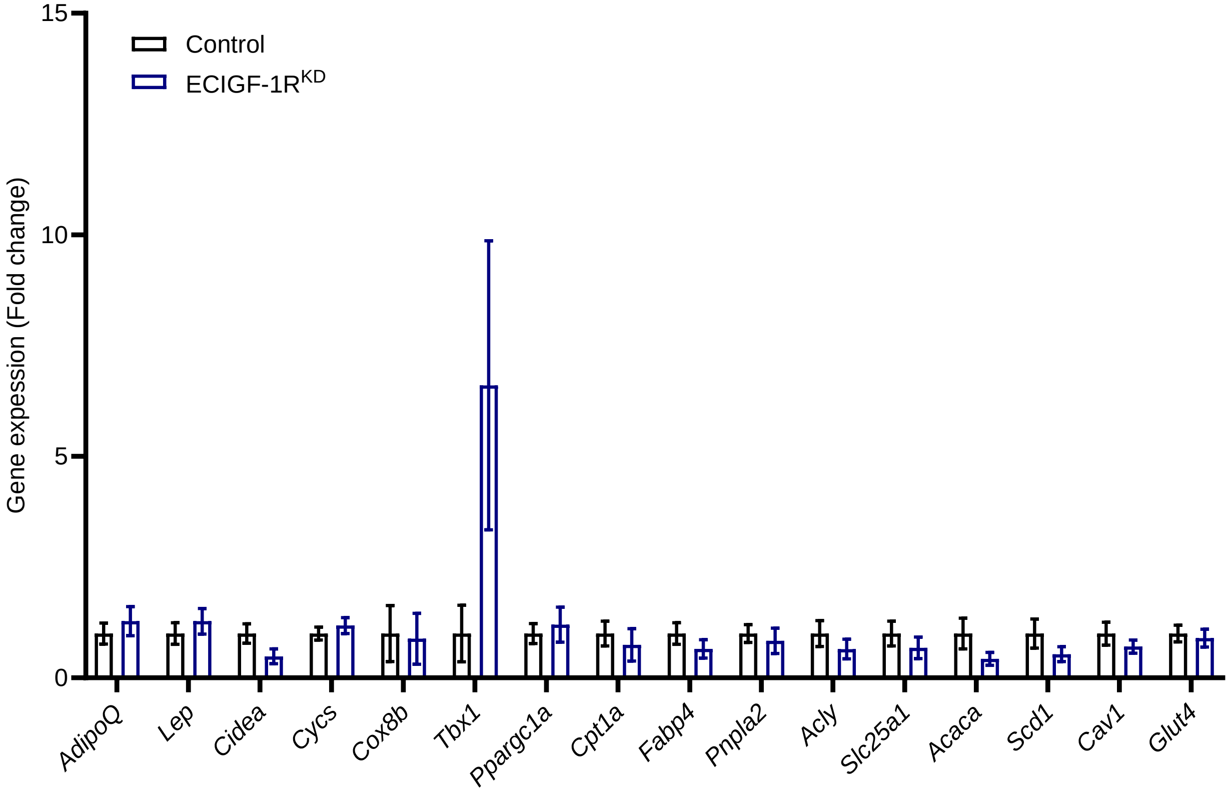

Supplementary figure 5 – White epididymal adipose gene expression in mice with reduced endothelial IGF-1R expression after 2 weeks of high fat diet

A. Quantification of epididymal white adipose tissue (eWAT) gene expression from 2-week high fat diet (HFD) control and tamoxifen-inducible endothelial cell specific IGF-1R knockdown mice ($ECIGF\text{-}1R^{KD}$) mice that were downregulated (n =9-17).

B. Quantification of eWAT gene expression from 2-week HFD control and $ECIGF\text{-}1R^{KD}$ mice that were unchanged (n =9-17).

Data shown as mean ± SEM, data points are individual mice. $p<0.05$ taken as being statistically significant using student unpaired two tailed t-test and denoted as *.

Supplementary Figure 6

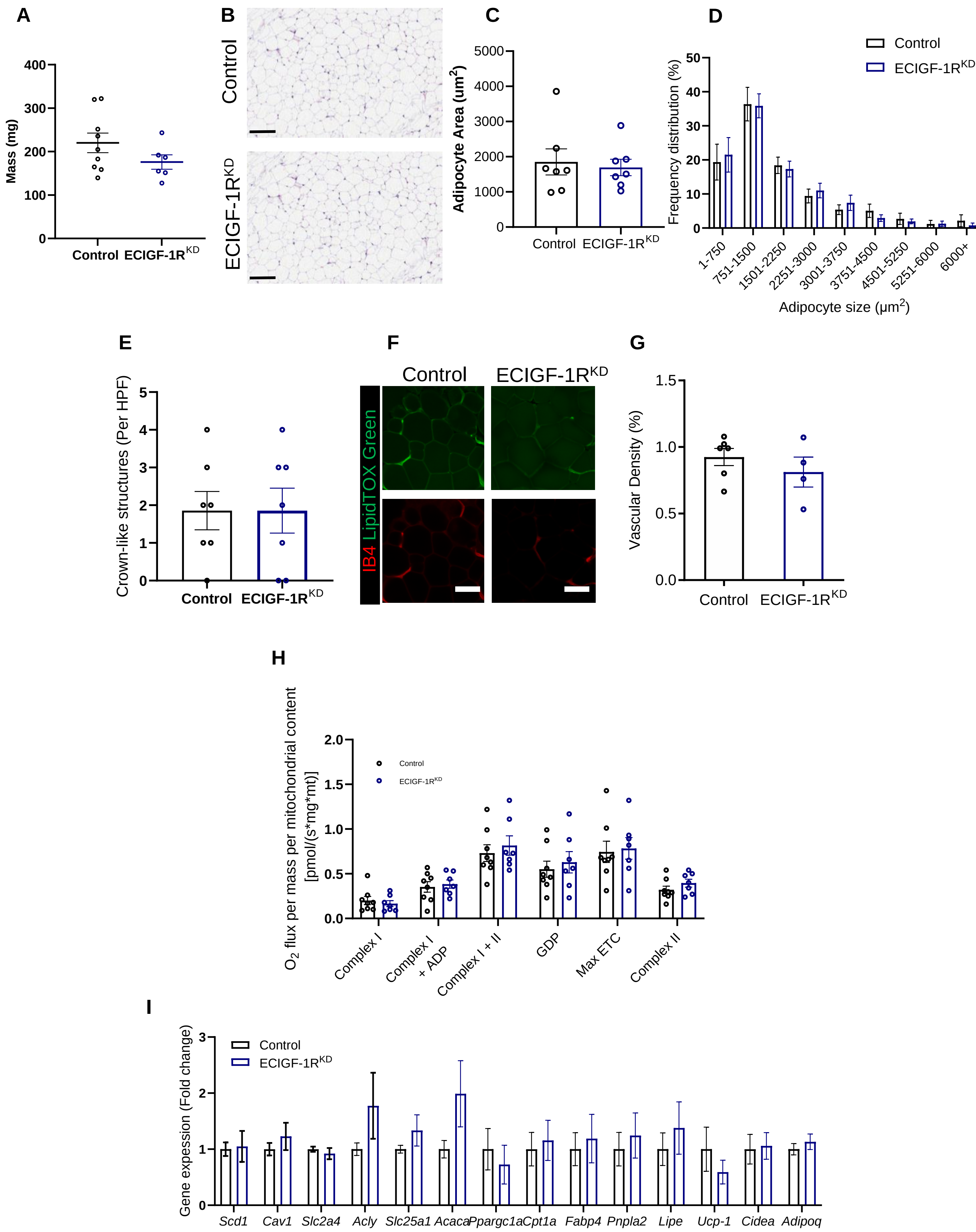

Supplementary figure 6 – Reduction in murine endothelial IGF-1R expression prevents deleterious remodelling of white adipose tissue in the setting of high fat feeding in a depot specific manner

A. Quantification of inguinal white adipose tissue (iWAT) mass from 2-week high fat diet (HFD) control and tamoxifen-inducible endothelial cell specific IGF-1R knockdown mice (ECIGF-1R^{KD}) mice. (n=6&9).

B. Representative images of hematoxylin and eosin (H & E) stained iWAT from 2-week HFD control and ECIGF-1R^{KD} mice (Scale bar = 100µm).

C. Quantification of adipocyte size in iWAT from 2-week HFD control and ECIGF-1R^{KD} mice (n =7&7).

D. Quantification of adipocyte size distribution in iWAT from 2-week HFD control and ECIGF-1R^{KD} mice (n =7&7).

E. Quantification of crown-like structures per high powered field (HPF) in iWAT from 2-week HFD control and ECIGF-1R^{KD} mice (n =5&6).

F. Representative images of isolectin B4 (Red) and LipidTox (Green) stained iWAT from 2-week HFD control and ECIGF-1R^{KD} mice (Scale bar =100 µm).

G. Quantification of iWAT vascularisation from 2-week HFD control and ECIGF-1R^{KD} mice (n =6&4).

H. Quantification of respiration from iWAT normalised to mitochondrial content from 2-week HFD control and ECIGF-1R^{KD} mice (n=7&8).

I. Quantification of iWAT gene expression from 2-week HFD control and ECIGF-1R^{KD} mice (n =5-8).

Data shown as mean ± SEM, data points are individual mice. $p<0.05$ taken as statistically significant using student unpaired two-tailed t-test and denoted as * (**$p \leq 0.01$). For respirometry data, two-way ANOVA was corrected for multiple comparisons by controlling the False Discovery Rate using the two-stage step-up method of Benjamani, Krieger, and Yekutieli.

Supplementary Figure 7

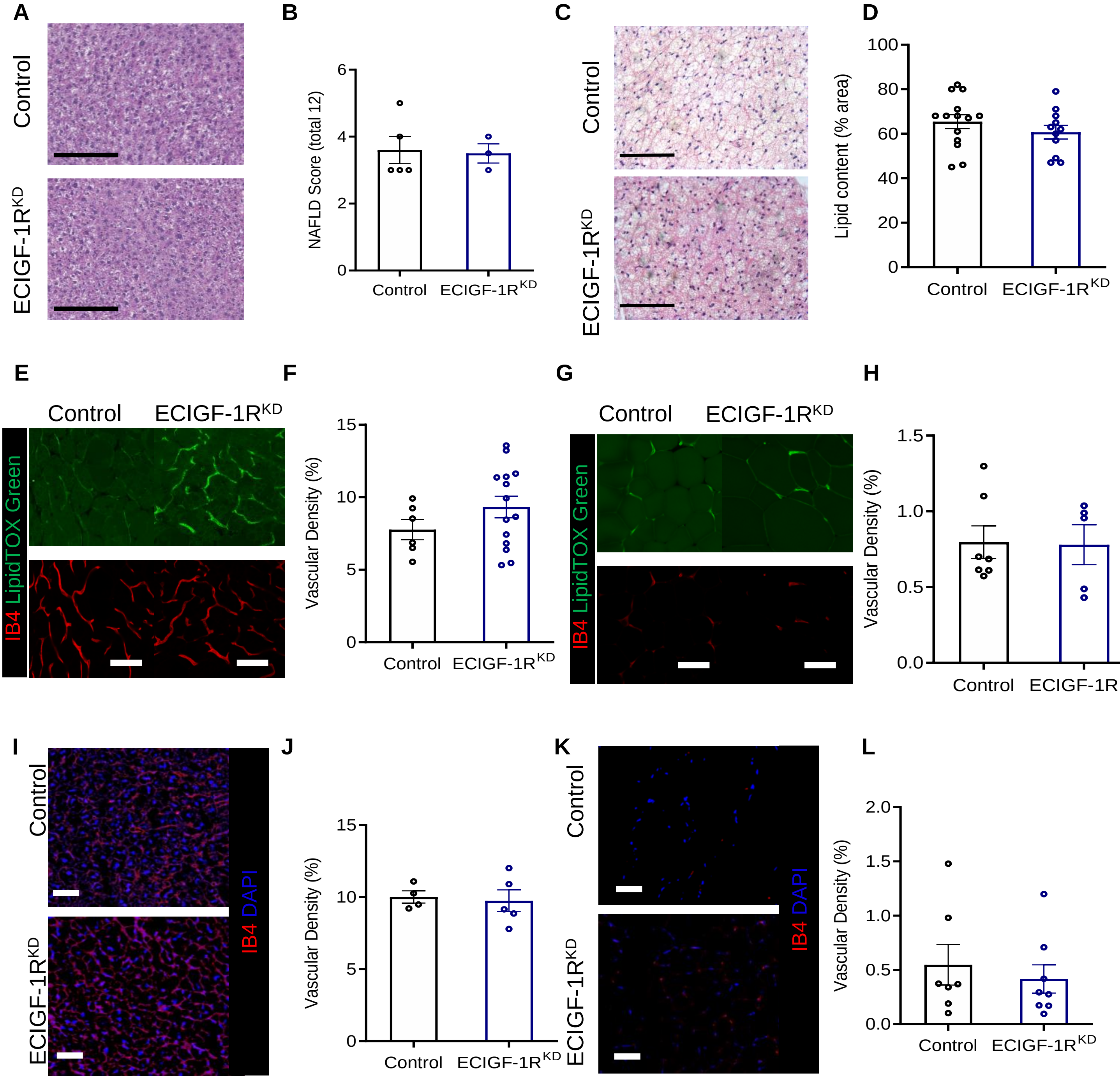

Supplementary figure 7 – Histological characterisation of mice with reduced endothelial IGF-1R expression after 2 weeks of high fat diet

A. Representative images of hematoxylin and eosin (H and E)-stained liver from 2-week high fat diet (HFD) control and tamoxifen-inducible endothelial cell specific IGF-1R knockdown mice ($ECIGF\text{-}1R^{KD}$) mice (Scale bar = 200µm).

B. Quantification of non-alcoholic fatty liver disease (NAFLD) from 2-week HFD control and $ECIGF\text{-}1R^{KD}$ mice (n =5&3).

C. Representative images of H and E-stained interscapular brown adipose tissue (BAT) from 2-week HFD control and $ECIGF\text{-}1R^{KD}$ mice (Scale bar = 100µm).

D. Quantification of lipid content of BAT from 2-week HFD control and $ECIGF\text{-}1R^{KD}$ mice (n =14&12).

E. Representative images of isolectin B4 (Red) and LipidTox (Green) stained BAT from 2-week HFD control and $ECIGF\text{-}1R^{KD}$ mice (Scale bar = 100µm).

F. Quantification of vascularity in interscapular brown adipose tissue from 2-week HFD control and $ECIGF\text{-}1R^{KD}$ mice (n =6&14).

G. Representative images of isolectin B4 (Red) and LipidTox (Green) stained perinephric white adipose tissue from 2-week HFD control and $ECIGF\text{-}1R^{KD}$ mice (Scale bar = 100µm).

H. Quantification of vascularity in perinephric white adipose tissue from 2-week HFD control and $ECIGF\text{-}1R^{KD}$ mice (n =7&5).

I. Representative images of isolectin B4 (Red) and DAPI (Blue) stained liver from 2-week HFD control and $ECIGF\text{-}1R^{KD}$ mice (Scale bar = 50µm).

J. Quantification of liver vascularisation from 2-week HFD control and $ECIGF\text{-}1R^{KD}$ mice (n =4&5).

K. Representative images of isolectin B4 (Red) and DAPI (Blue) stained muscle from 2-week HFD control and $ECIGF\text{-}1R^{KD}$ mice (Scale bar = 50µm).

L. Quantification of muscle vascularisation from 2-week HFD control and $ECIGF\text{-}1R^{KD}$ mice (n =7&8).

Data shown as mean ± SEM, Individual mice are shown as separate data points $p<0.05$ taken as being statistically significant using a student unpaired two tailed t-test and denoted as *.

Supplementary Figure 8

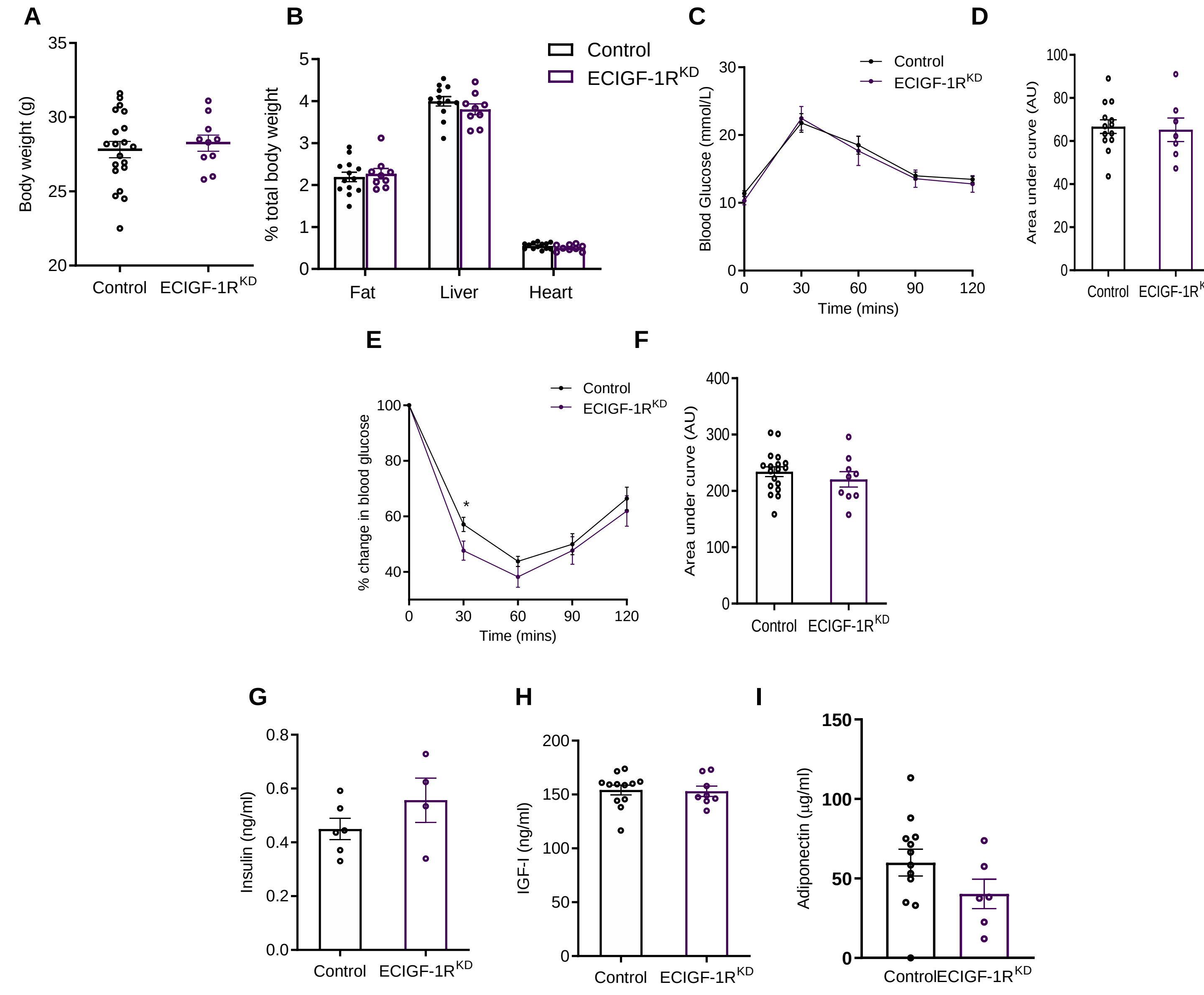

Supplementary figure 8 – Metabolic characterisation of mice with reduced endothelial IGF-1R expression after 8-weeks high fat diet.

A. Body mass of 8-week high fat fed (HFD) fed control and tamoxifen-inducible endothelial cell specific IGF-1R knockdown mice (ECIGF-1R^{KD}) mice (n=20&10).

B. Wet organ weight of 8-week HFD control and ECIGF-1R^{KD} mice (n=13&9).

C. Glucose tolerance over time of 8-week HFD fed control and ECIGF-1R^{KD} mice (n=13&7).

D. Area under the curve (AUC) analysis of glucose tolerance of 8-week HFD fed control and ECIGF-1R^{KD} mice (n=13&7).

E. Insulin tolerance over time of 8-week HFD fed control and ECIGF-1R^{KD} mice (N=18&11).

F. Area under the curve (AUC) analysis of insulin tolerance test of 8-week HFD fed control and ECIGF-1R^{KD} mice (n=18&11).

G. Fasting plasma insulin levels from 8-week HFD fed control and ECIGF-1R^{KD} mice (n=6&4).

H. Fasting plasma IGF-1 levels from 8-week HFD fed control and ECIGF-1R^{KD} mice (n=12&8).

I. Fasting plasma adiponectin levels from 8-week HFD fed control and ECIGF-1R^{KD} mice (n=12&8).

Data shown as mean ± SEM, individual mice are shown as separate datapoints $p<0.05$ taken as being statistically significant using a student unpaired two tailed t-test and denoted as *.

Supplementary Figure 9

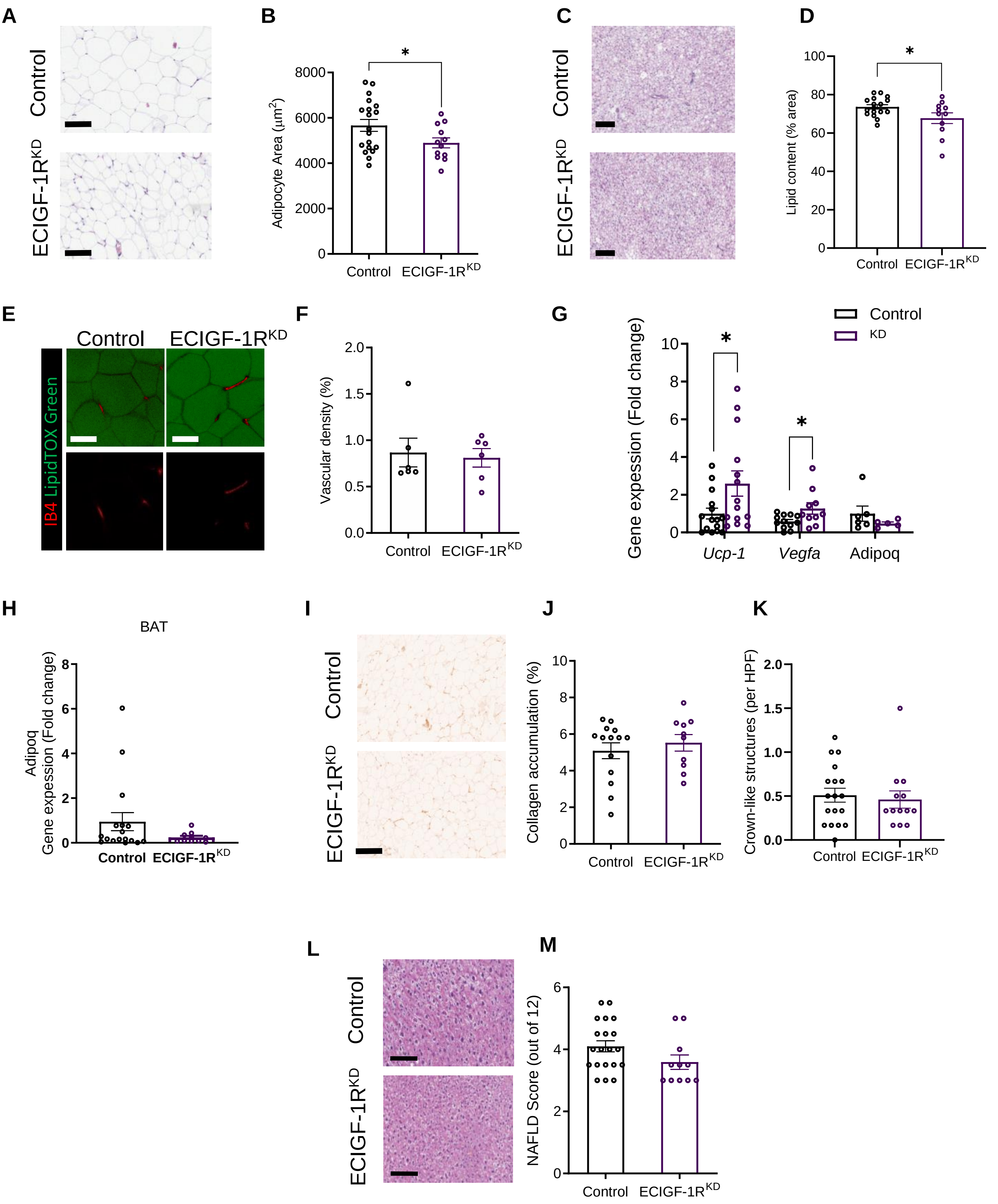

Supplementary figure 9 – Histological characterisation of mice with reduced endothelial IGF-1R expression after 8-weeks high fat diet.

A. Representative images of hematoxylin and eosin (H & E) stained epididymal white adipose (eWAT) tissue from 8-week high fat diet (HFD) fed control and tamoxifen-inducible endothelial cell specific IGF-1R knockdown mice (ECIGF-1R^{KD}) mice (Scale bar = 200μm).

B. Quantification of eWAT of adipocyte size from 8-week HFD fed control and ECIGF-1R^{KD} mice (n= 12&18).

C. Representative images of H & E stained interscapular brown adipose tissue (BAT) from 8-week HFD fed control and ECIGF-1R^{KD} mice (Scale bar = 200μm).

D. Quantification of lipid content of BAT from 8-week HFD fed control and ECIGF-1R^{KD} mice (n =18&13).

E. Representative images of isolectin B4 (Red) and LipidTox (Green) stained eWAT from 8-week HFD control and ECIGF-1R^{KD} mice (Scale bar = 100μm).

F. Quantification of eWAT vascularisation from 8-week HFD fed control and ECIGF-1R^{KD} mice (n=6&6).

G. Quantification of eWAT gene expression of *Ucp-1*, *Vegfa* and *Adipoq* from 8-week HFD fed control and ECIGF-1R^{KD} mice. (n=12-15).

H. Quantification of BAT gene expression of *Adipoq* from 8-week HFD fed control and ECIGF-1R^{KD} mice. (n=12-15).

I. Representative images of picro sirius red stained eWAT from 8-week HFD fed control and ECIGF-1R^{KD} mice (Scale bar = 200μm).

J. Quantification of eWAT collagen deposition from 8-week HFD fed control and ECIGF-1R^{KD} mice (n=14&10).

K. Quantification of eWAT crown like structures from 8-week HFD fed control and ECIGF-1R^{KD} mice (n=13&17).

L. Representative images of H and E-stained liver from 8-week HFD fed control and ECIGF-1R^{KD} mice (Scale bar = 200μm).

M. Quantification of non-alcoholic fatty liver disease (NAFLD) from 8-week HFD fed control and ECIGF-1R^{KD} mice (n=20&11).

Data shown as mean ± SEM, individual mice are shown as separate datapoints $p<0.05$ taken as being statistically significant using a student unpaired two tailed t-test and denoted as *.

Supplementary Figure 10

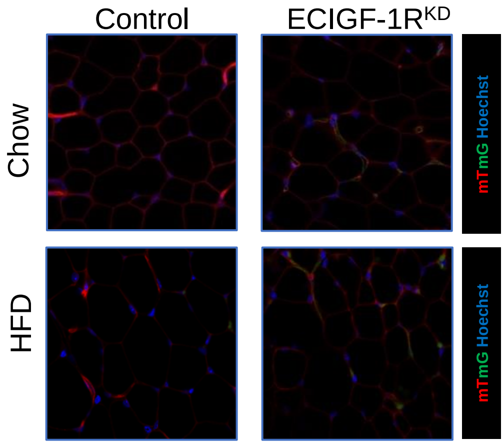

Supplementary figure 10 – Adipocytes from mice with reduction in endothelial IGF-1R expression are not from endothelial lineage

Following induction with tamoxifen, cells of endothelial cell lineage in the tamoxifen-inducible endothelial cell specific IGF-1R knockdown mice (ECIGF-1R^{KD}) mice fluorescent green using the mTmG system, with all other cells fluorescing red. All adipocytes from both genotypes appear red, confirming adipocytes are not from endothelial lineage.

Supplementary Figure 11

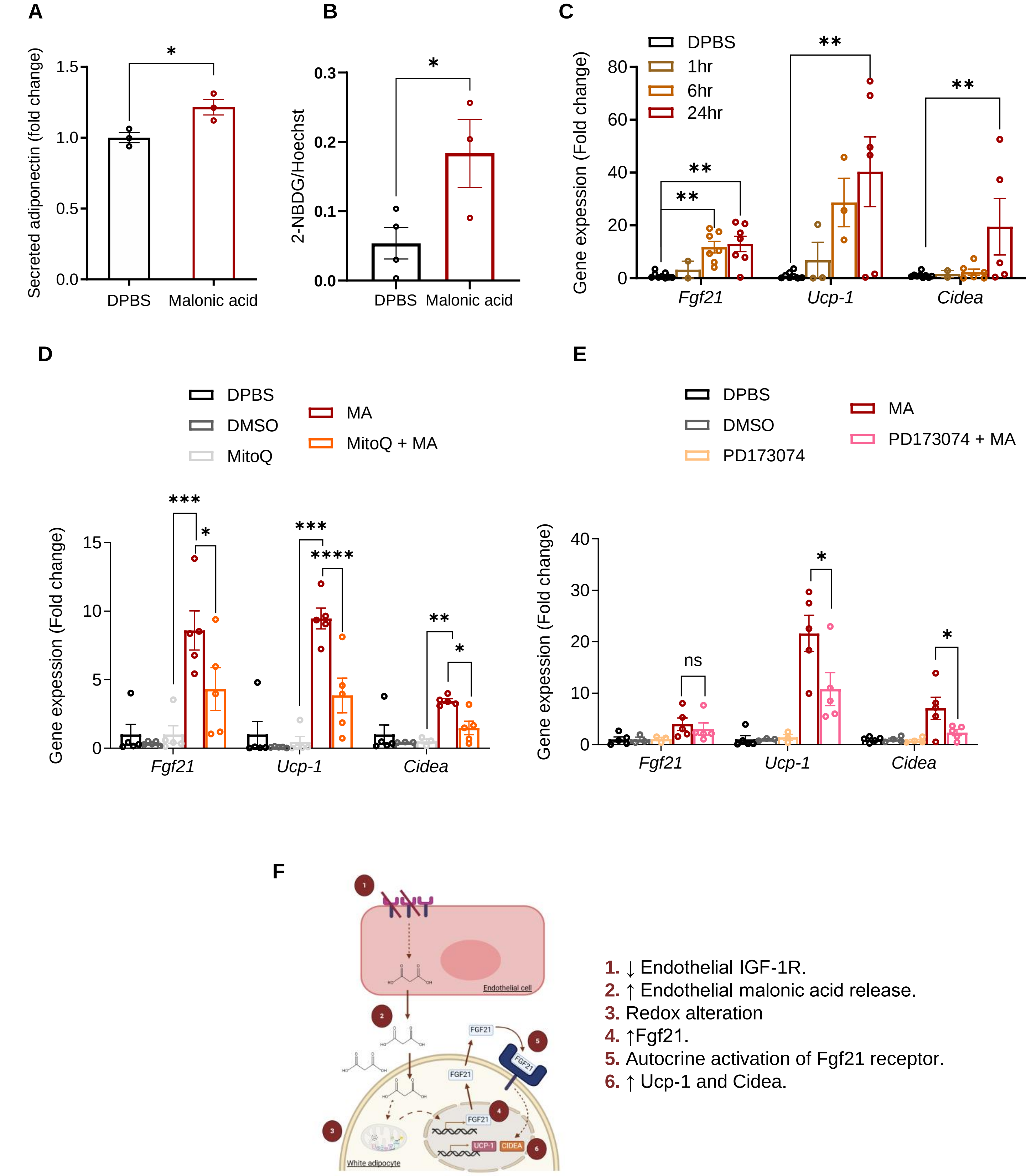

Supplementary figure 11 – A role for malonate in modulating adipose function

A. Quantification of adiponectin secretion in 3T3-L1 adipocytes after 24hr 10mM malonic acid stimulation (n =3 per treatment group).

B. Quantification of glucose uptake in 3T3-L1 adipocytes after 24hr 10mM malonic acid stimulation (n =3-4 per treatment group).

C. Quantification of gene expression in 3T3-L1 adipocytes after varying exposure times to 10mM malonic acid stimulation (n =4-7 per treatment group).

D. Quantification of *Fgf21, Ucp-1 and Cidea* gene expression in 3T3 -L1 adipocytes, after treatment with mitoQ and malonic acid (MA) 10mM for 24hrs (n =5 per treatment group).

E. Quantification of *Ffg21, Ucp-1* and *Cidea* gene expression in 3T3-L1 adipocytes after treatment with FGF1R blocker (PD17304) and malonic acid 10mM for 24hrs (n =3-5 per treatment group).

F. Schematic diagram of the proposed mechanism of endothelial cell specific IGF-1R mediated white adipocyte modulation. Created in BioRender. Luk, C. (2021) https://BioRender.com/n49y502 .

Data shown as mean ± SEM, n is an individual experiment. $p<0.05$ taken statistically significant using student unpaired two tailed t-test or ANOVA and denoted as * ($p \le 0.01$ and is denoted as **).

Supp Table 1: Primers for qPCR

Gene	Bio-rad Assay code
**Adipoq**	qMmuCED0045486
**Acaca**	qMmuCID0006041
**Acly**	qMmuCID0021118
**Cav1**	qMmuCID0020997
**Cidea**	qMmuCID0007140
**Cd137**	qMmuCED0047964
**Cited1**	qMmuCED0037644
**Cpt1a**	qMmuCID0021095
**Cox8b**	qMmuCID0020689
**Cycs**	qMmuCED0001027
**Fabp4**	qMmuCID0007422
**Fgf21**	qMmuCED0025797
**Gapdh**	qMmuCED0027497
**Glut4**	qMmuCED0024734
**Lep**	qMmuCID0040177
**Ppargc1α**	qMmuCID0006032
**Pparα**	qMmuCED0046526
**Ppary**	qMmuCID0018821
**Pnpla2**	qMmuCED0004671
**Pln1**	qMmuCID0005354
**Nrg4**	qMmuCID0024409
**Tbx1**	qMmuCID0011851
**Tmem26**	qMmuCED0061015
**Slc25a1**	qMmuCED0003920
**Slc2a4**	qMmuCED002473
**Scd1**	qMmuCID0011348
**Ucp1**	qMmuCED0047500
**Vegfa**	qMmuCED0040260
**CD137**	qHsaCID0020895
**CIDEA**	qHsaCED0003559
**CYCS**	qHsaCED0046874
**FGF21**	qHsaCIP0032896
**GAPDH**	qHsaCED0038674
**PPARGC1α**	qHsaCID0006418
**TMEM26**	qHsaCID0009380
**UCP-1**	qHsaCED0043275
